# Dopamine Supersensitivity: A Novel Hypothesis of Opioid-Induced Neurobiological Mechanisms Underlying Opioid-Stimulant Co-use and Opioid Relapse

**DOI:** 10.3389/fpsyt.2022.835816

**Published:** 2022-04-15

**Authors:** Justin C. Strickland, Cassandra D. Gipson, Kelly E. Dunn

**Affiliations:** ^1^Department of Psychiatry and Behavioral Sciences, Johns Hopkins University School of Medicine, Baltimore, MD, United States; ^2^Department of Pharmacology and Nutritional Sciences, University of Kentucky, Lexington, KY, United States

**Keywords:** opioid, stimulant, treatment, methamphetamine, relapse, withdrawal, cocaine

## Abstract

Emergent harms presented by the co-use of opioids and methamphetamine highlight the broader public health challenge of preventing and treating opioid and stimulant co-use. Development of effective therapeutics requires an understanding of the physiological mechanisms that may be driving co-use patterns, specifically the underlying neurobiology of co-use and how they may facilitate (or be leveraged to prevent) continued use patterns. This narrative review summarizes largely preclinical data that demonstrate clinically-meaningful relationships between the dopamine and opioid systems with direct implications for opioid and stimulant co-use. Synthesized conclusions of this body of research include evidence that changes in the dopamine system occur only once physical dependence to opioids develops, that the chronicity of opioid exposure is associated with the severity of changes, and that withdrawal leaves the organism in a state of substantive dopamine deficit that persists long after the somatic or observed signs of opioid withdrawal appear to have resolved. Evidence also suggests that dopamine supersensitivity develops soon after opioid abstinence and results in increased response to dopamine agonists that increases in magnitude as the abstinence period continues and is evident several weeks into protracted withdrawal. Mechanistically, this supersensitivity appears to be mediated by changes in the sensitivity, not quantity, of dopamine D2 receptors. Here we propose a neural circuit mechanism unique to withdrawal from opioid use with implications for increased stimulant sensitivity in previously stimulant-naïve or inexperienced populations. These hypothesized effects collectively delineate a mechanism by which stimulants would be uniquely reinforcing to persons with opioid physical dependence, would contribute to the acute opioid withdrawal syndrome, and could manifest subjectively as craving and/or motivation to use that could prompt opioid relapse during acute and protracted withdrawal. Preclinical research is needed to directly test these hypothesized mechanisms. Human laboratory and clinical trial research is needed to explore these clinical predictions and to advance the goal of developing treatments for opioid-stimulant co-use and/or opioid relapse prevention and withdrawal remediation.

## Introduction

Historically, there have been periods of time in which the co-use of opioids and stimulants has been highly prevalent and of significant public health concern. The frequency of opioid-stimulant co-use has tended to wax and wane over the years and in the past decade the primary public health concern has focused on exclusive opioid use. However, now, amidst the ongoing opioid epidemic, this opioid-stimulant polysubstance use trend has reemerged. Deemed a “Fourth Wave” or “Twin Epidemic,” epidemiological evidence now emphasizes a renewed and rapidly increasing public health harm of concurrent stimulant use, particularly methamphetamine use, among people who use opioids (1, 2). National and regional treatment admission data report stark increases in recent methamphetamine use among people entering treatment for opioid use disorder (OUD) (3–5), representing an approximate 5-fold increase in methamphetamine use among primary heroin treatment admissions from 2008 to 2017 (3). Such trends are also evident in national prevalence data (6–8). Data from the nationally representative National Survey on Drug Use and Health (NSDUH), for instance, show that past month methamphetamine use increased five-fold from 9% in 2015 to 44% in 2019 among people who also used heroin in the past month (6). Related surges in methamphetamine-involved overdoses in combination with opioids have been observed (9–13) with greater increases in non-cocaine psychostimulant overdoses in states with a greater prevalence of opioid use disorder (OUD) (12). This concomitant use of opioids and methamphetamine is worrisome beyond this noted overdose risk given other associations with psychiatric comorbidity, infectious disease transmission, and healthcare utilization (6, 14, 15). Moreover, although treatments for opioids and opioid overdose exist, no such treatments are available for stimulants, suggesting that the population of persons with co-use may face significant challenges to recovery.

These emergent concerns underscore broader challenges presented by opioid and dopamine agonist (“stimulant”) co-use. While recent public health emphasis has been placed on opioids and methamphetamine, the practice of opioid-stimulant co-use dates back decades with trends observed across diverse subgroups and geographic regions. The co-use of opioids and cocaine, for example, was extensively described throughout the 1980's, 90's, and 00's in the United States [e.g., (16, 17)] and more globally [e.g., (18, 19)]. Reports of simultaneous (i.e., “speedballs”) or concurrent co-use of opioids and cocaine motivated intense preclinical and clinical investigation into novel treatments (20). Despite these efforts, opioid and stimulant co-use remains a challenging treatment phenomenon with no FDA approved medication for co-use and weak to negative evidence for those pharmacotherapies that have been tested [e.g., (21)]. Development of effective therapeutics requires an understanding of the mechanisms driving co-use, specifically the underlying neurobiology of co-use and how these neurobiological mechanisms may facilitate (or be leveraged to prevent) continued use patterns.

### Goal of This Review

The purpose of this review is to synthesize data collected primarily from preclinical studies dating back to the 1950's that demonstrate clear relationships between the dopamine and opioid systems with direct implications for opioid and stimulant co-use. These data outline a hypothetical but mechanistically-based premise for why opioid and stimulant co-use occurs. Notably, this hypothesis pertains specifically to the onset of stimulant use in persons who have opioid physical dependence. This is not meant to suggest that persons who are co-using these substances were naïve to stimulants prior to using opioids, rather the following conversation focuses on the large proportion of persons whose most recent use period was not characterized by concurrent initiation of opioids and stimulants together but rather is characterized by a new stimulant use episode that begins after opioid physical dependence has developed. This is a relatively common pattern that has been evident for several decades, most notably in persons who are receiving methadone for opioid use disorder treatment (22–25), for which numerous interventions have been evaluated to address new stimulant use (21, 26–29). In effect, this review is proposing a novel mechanistic hypothesis that the development of opioid physical dependence changes underlying neurobehavioral mechanisms in such a way that the experience of stimulants is uniquely different from that prior to opioid dependence development.

This hypothesis also has implications for the treatment of OUD, particularly relapse to opioids, and we have therefore outlined a putative and testable underlying neurobiological mechanism we hypothesize may function as a barrier to the development of effective opioids use disorder treatments. The data reviewed here are primarily drawn from preclinical animal studies; this hypothesis has not been prospectively examined in human subjects. Thus, the limited human laboratory, clinical, and qualitative studies available in this area are also reviewed to provide corroborating preliminary evidence for these mechanistic predictions in support of more focused prospective research.

Specifically, we propose a novel mechanism involving enhanced dopamine D2 receptor-mediated activity of the striatal-ventral mesencephalon-thalamic circuit, which we propose occurs as a function of chronic opioid exposure and results in organisms that are being withdrawn from opioids having a unique dopaminergic experience. We suspect these conformational changes may cause dopamine agonists to take on enhanced reinforcing properties during states of acute or protracted opioid withdrawal, including stimulants that are introduced after opioid physical dependence has been developed. This review is meant to present a novel yet testable hypothesis that has not yet been examined in human subjects and which has the potential to yield insights that could contribute meaningfully to collective efforts to address opioid and stimulant co-use. Thus, this review concludes with directions for future work to address the sustained morbidity and mortality presented by the co-use of opioids and stimulants.

## Brief Overview of Opioid and Dopamine Neurobiology

Prior reviews have discussed the relationship between opioid and dopamine neurobiology and its relevance for opioid use and OUD [see contemporary and classic reviews in (30, 31)], so these concepts are reviewed here only briefly to support interpretation of the summarized results. The opioid system is regarded as the natural analgesia system and is distributed throughout the central and peripheral nervous systems. Opioids, such as morphine, oxycodone, and heroin, function as agonists that bind to the opioid mu, kappa, and delta (as well as ORL-1 and nociception/orphanin) receptors. The strength of conventional opioid effects (e.g., analgesia, euphoria) are primarily related to the strength of activity the opioid confers on the mu opioid receptor. The dopamine system is widely regarded as the primary reward and motivation system that is responsible for producing euphoria and for reinforcing repeated drug use behavior. Dopamine neurons are highly concentrated in the midbrain, which is characterized by projections from the ventral tegmental area (VTA) to the nucleus accumbens in the striatum (i.e., the mesolimbic system) or to the prefrontal cortex (i.e., the mesocortical system). The degree to which this system is activated corresponds generally to the degree of reward experienced. Additional and important nuances also exist with regard to the dopamine receptor system, which are categorized into D1 and D2 families. D1 family receptors (D1 and D5 receptor subtypes) are Gs-coupled receptors that generally produce excitatory signals; D2 family receptors (D2, D3, and D4 receptor subtypes) are Gi-coupled receptors that generally produce inhibitory signals. Moreover, when D2 family receptors are found presynaptically they often function as autoreceptors that regulate (e.g., inhibit) dopamine release and firing (32). The nucleus accumbens contains both D1 and D2 receptor families of receptors.

Although opioids exert their primary effects via agonism of the mu opioid receptor, these drugs also exert indirect effects on the mesocorticolimbic dopamine system (33–35). Mu opioid receptors within the VTA are located on GABAergic interneurons, which reside within the VTA (33–35). Under drug naïve conditions, these GABAergic cells provide inhibitory tone on dopamine neurons, which project to the nucleus accumbens. Within the nucleus accumbens, dopamine provides modulatory tone on GABAergic medium spiny neurons (MSNs), which express either D1 or D2 receptors. In preclinical studies, MSNs within the nucleus accumbens are critical in driving use of drugs, including opioids. Importantly, this has been shown to be driven by D1-expressing and not D2-expressing MSNs, as most of these cells express either D1 or D2 and have been heavily studied for their opposing roles in substance use (36). Thus, the recent literature regarding D1 versus D2 supports an important role of D1-expressing MSNs in regulating stimulant use, whereas D2-expressing MSNs are involved in negative regulation of these behaviors (36–39). As well, there is a large body of literature outlining the output structures of these differential cell populations [e.g., (40–42)]. Although emerging evidence suggests that reinstatement to heroin-associated cues induces synaptic adaptations at D1-expressing MSNs within the nucleus accumbens (43), it is not clear if the outcome measure of matrix metalloproteinase activity surrounding D1 or D2 synapses captures differences in sensitivity of these different dopamine receptor subtypes to subsequent dopamine agonism.

It is important to note that a large number of preclinical studies examining the impacts of D1 vs. D2 pathways on psychostimulant or opioid-related behaviors have generally studied this in animals under protracted withdrawal from these drugs and there may be adaptations specific to drug taking vs. withdrawal. When opioids are present (either systemically administered or locally applied *in vitro*), prior studies show an inhibition of the firing rate of VTA GABA neurons (44, 45), and the canonical pathway would indicate that this then reduces GABAergic inhibition of accumbotegmental dopamine cells (i.e., cells that project from the VTA to the nucleus accumbens), ultimately leading to an increase in dopamine signaling within the nucleus accumbens (44) and, subsequently, an increase of dopamine receptor activation on nucleus accumbens MSNs. It is thought that this neural mechanism contributes to the classic euphoric response produced by opioid drugs. However, one cardinal study showed that selective ablation of dopamine terminals in the nucleus accumbens induced long-lasting reductions in cocaine but not heroin self-administration (46), suggesting that other neurotransmitter systems beyond dopamine signaling are involved in the reinforcing effects of opioids.

**Figure 1:**
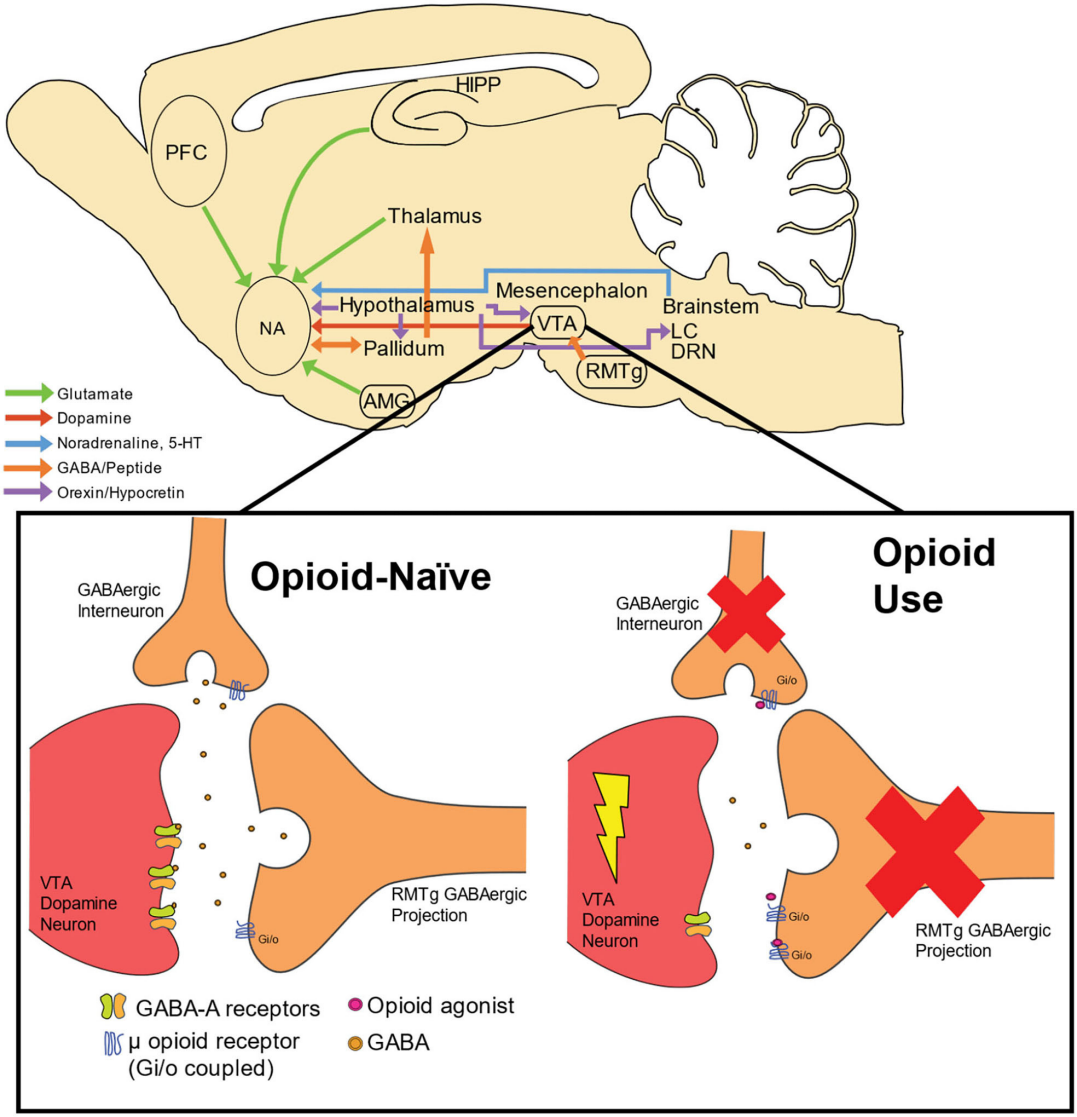
Neural circuitry and dopamine disinhibition by opioid use. Neural circuitry involved in opioid use includes cortical, striatal, thalamic, mesencephalon, and brainstem structures. Dopamine cell bodies residing within the VTA receive GABAergic innervation from both GABAergic interneurons and projection neurons from the RMTg. GABA activates GABA-A receptors located on dopamine neurons, thus providing inhibition of dopamine neuronal activity. Through these terminals, dopamine excitability is maintained in homeostasis within an opioid-naïve system. When opioids are present, these compounds act as agonists at inhibitory (G_i/o_) μ opioid receptors, which exerts inhibitory tone on GABAergic terminals synapsing onto dopamine cells. The net result is an enhancement of phasic dopamine release into terminal structures, including the NA. PFC, prefrontal cortex; HIPP, hippocampus; NA, nucleus accumbens, VTA; ventral tegmental nucleus; RMTg, rostromedial tegmental nucleus; LC, locus coeruleus; AMG, amygdala; DRN, dorsal raphe nucleus; 5-HT, serotonin.

In contrast to opioids, stimulant drugs produce their euphoric and reinforcing response by directly activating dopaminergic signaling within the reward pathway. Specifically, they are able to prolong the duration of time that dopamine can exert an effect on receptors by either preventing it from being recycled back into the neuron (e.g., cocaine) and/or by releasing large quantities of dopamine into the synapse (e.g., amphetamine or methamphetamine) (47). Another relatively recent piece of the circuit puzzle regarding stimulants and opioids involves the rostromedial tegmental nucleus (RMTg) or the “tail of the VTA” (tVTA), which project dense inhibitory tone to midbrain dopamine neurons. Importantly, the RMTg projects GABAergic tone into the VTA, and are generally thought to provide a “break” on motivated behavior (48). Bringing this newly charted neural circuit into focus with psychostimulant and opioid use, recent studies have found that the RMTg plays a critical role in aversive responses to cocaine (49), and acute withdrawal from cocaine increases cell firing within the RMTg (50). The RMTg also appears to be a critical mechanism in opioid-induced VTA dopamine disinhibition. As the canonical pathway, described above, typically considered GABAergic interneurons as the primary source of dopamine cell inhibition, one recent study showed that morphine induced a significant inhibition of inhibitory post-synaptic currents (IPSCs) evoked from the RMTg, whereas IPSCs evoked from VTA interneurons were almost insensitive to morphine (51). Taken together, these results support that the GABAergic projection from the RMTg is a critical, and perhaps dominant, neural circuit responsible for opioid disinhibition of dopamine neurons within the VTA. However, no study to date has examined this more recent circuit in the context of opioid and stimulant co-use. Figure 1 summarizes these neural circuits involved in opioid use as well as illustrates opioid-induced dopamine disinhibition in the VTA.

## Changes in the Dopamine System Following Chronic Opioid Exposure May Be Responsible for Opioid-Stimulant Co-Use and Stimulant Initiation

### Dopamine Is Meaningfully Involved With Opioid Effects and Opioid Withdrawal

Evidence that the dopamine system has meaningful interactions with the opioid system or expression of opioid effects have been reported as far back as 1954 (52). However, the manner through which this happens is nuanced. This is evident in a series of studies that revealed opioid agonists produce biphasic effects in animal models whereby low doses of opioids engender stimulant-like behavior, and the expected sedative-like effects of opioids are not elicited until higher doses are administered. In addition, in these studies tolerance to the depressant-like effects of opioids was observed to develop quickly over time, coincident with development of opioid physical dependence, whereas tolerance to the stimulant-like effects was not observed to develop at the same rate. In fact, continuous exposure to opioids was found to increase the emission of stimulant-like behaviors over time. These effects were firmly related to opioid activation because the stimulant-like behaviors produced by opioids can be blocked through administration of the opioid antagonist naloxone, demonstrating a causal relationship with opioid administration and opioid-receptor activity in the expression of behavior. The opioid-induced stimulation observed was only surmounted once large doses of opioids were administered in a repeated fashion (53–56), see also (57). The simulating effects of opioids have also been reported by human subjects, though this has only been examined in a small number of largely non-empirical studies (described below). The mesolimbic dopamine system appears to be a major contributor to the manifestation of opioid-induced stimulating effects (54, 58–60), and a convergence of data has also reliably implicated the dopamine system in the expression of some opioid withdrawal symptoms [see (61) for review], an effect that is especially profound with regard to thermoregulatory behavior (62).

As outlined above, it is well-established that exposure to opioids increases dopamine release in the striatum, and this is often hypothesized to be the mechanistic basis by which opioid-seeking behavior develops. However, there has been less discussion paid to the role dopamine may play during states of acute or prolonged opioid abstinence in animals that have developed opioid physical dependence. Several studies have revealed that when animals are made physically dependent on opioids and undergo withdrawal that is either spontaneous in nature (e.g., discontinuation of opioid agonists) or precipitated by administration of an opioid antagonist (e.g., naloxone, naltrexone), dopamine levels in the striatum decrease. This is evident through multiple different assays, including microdialysis quantification of extracellular dopamine levels (63–67), analysis of striatal brain tissue (68, 69), morphological examination of dopamine-containing neurons (70), *in situ* hybridization quantification of striatal adenylate cyclase levels (71), and 6-hydroxydopamine (6-OHDA) lesioning assays (72). The decrease observed in dopamine signaling during a state of abstinence is not simply a function of having been recently exposed to an opioid agonist because such changes do not occur during periods of acute opioid agonist exposure and the level of dopamine depletion that occurs has been correlated with the somatic expression of withdrawal (63, 66, 67). Moreover, the decrease in dopamine observed in animals that have opioid physical dependence and are put into a state of opioid abstinence is substantial, ranging from 25 to 35% of the level observed in control animals (67, 73). We know of only one human study that has examined this effect. That study used positron emission tomography (PET) to compare dopamine release in persons with OUD during a state of naloxone-precipitated withdrawal vs. a state of satiety. The study found that withdrawal was associated with a rapid and significant release of dopamine in the striatum and that the degree to which subjects reported the withdrawal to be aversive correlated with the strength of the dopamine release (74).

Conformational changes in dopamine signaling are also evident via electrophysiological assays. For instance, neuronal recordings of spontaneous meso-accumbens dopaminergic activity have revealed that rats that are made physically-dependent on morphine and then withdrawn exhibit reduced dopamine firing rates relative to control animals, and that both gross and burst firing rates continue to be low when measured 24-h after the final morphine exposure. The same effect was observed when opioid withdrawal was precipitated with a naloxone injection. The reduction in neuronal firing rates could also be reversed by intravenous administration of morphine, which was found to restore dopamine firing rates to the levels observed in control animals (75). Importantly, these changes in firing patterns only became evident when animals underwent a long period of abstinence (24 h); no such differences were observed when the animal was tested after 2 h of abstinence (75). The fact that this effect is easily reversed through provision of an opioid demonstrates a causal relationship between a state of abstinence and change in dopamine firing patterns. Another study that used microdialysis to examine postsynaptic dopamine levels found a similar effect. In that study, rats that underwent spontaneous withdrawal from opioids for 1 day evidenced levels of striatal dopamine that were 80% lower than control animals, and a dose of morphine was found to decrease this gap in a dose-dependent manner but did not fully restore the levels to those observed in control animals. In contrast, rats that were spontaneously withdrawn from opioids and left untreated continued to demonstrate lower striatal dopamine levels than controls for up to 3 days (the longest time frame examined in this study) (63). A follow-up microdialysis examination of mesolimbic dopamine levels in rats withdrawn from opioids found that extracellular dopamine levels were decreased in animals as far out as 7 days after the final opioid administration (76).

### Changes in the Dopamine System Remain Evident Long After the Somatic Signs of Opioid Withdrawal Have Remitted

Changes in dopamine signaling have been observed to persist for several days after the somatic signs of withdrawal appeared to have remitted, suggesting that the animals are continuing to experience an altered dopaminergic state even when overt signs of withdrawal are not apparent. This has been demonstrated with microdialysis, which revealed that animals that were withdrawn from opioids showed reduced rates of striatal dopamine levels even after the signs of withdrawal remitted (63). A second study reported that rats withdrawn from opioids showed observable somatic signs of withdrawal until around day 3 of abstinence, yet the electrophysiological reduction observed in their dopamine firing rates were pronounced up to day 7 and only showed signs of full resolution around day 14 of abstinence. When morphine was administered to those animals on day 14, their striatal dopamine levels surged well-beyond the levels observed in the control animals, suggesting they had entered a state of dopamine supersensitivity (77).

### The D2 Family of Receptors May Be Responsible for Enduring Changes in the Dopamine System Once Opioid Physical Dependence Is Established

Growing evidence has implicated the D2 family of receptors in the altered dopaminergic state that is produced by chronic opioid exposure. For instance, *in situ* hybridization of D1 and D2 receptor mRNA in rats that were made physically dependent on morphine showed that chronic opioid exposure increased only D2 receptor mRNA levels. These changes were specifically observed in the nucleus accumbens and striatum, which increased by as much as 27% relative to controls; no effect was observed with D1 receptors (78). Data from genetically-modified mice provide additional insight into this process by suggesting that the involvement of the D2 receptor becomes relevant only once physical dependence is developed. In this study, mice that were genetically engineered to be D2 (+/+) or D2 (-/-) were both able to develop morphine physical dependence and shows signs of withdrawal following naloxone administration. However, although D2 (+/+) mice showed conditioned place aversion to environments in which naloxone was administered, the D2 (-/-) mice showed no such aversion. Comparisons to opioid naïve mice further suggested that the D2 receptor was crucial for maintaining opioid motivation but only once the animal developed opioid physical dependence and was in a state of withdrawal, and that D2 was not implicated in behavior when the animal was opioid naïve and/or developing opioid-use behaviors (79).

Limited research has empirically examined this concept in humans. One study used a combined positron emission tomography (PET) imaging and drug administration study in adults who did (*n* = 16) and did not (*n* = 16) have a history of heroin use. Data revealed that adults who had used heroin showed reduced D2 family receptor availability and presynaptic dopamine release. However, neither of those outcomes were significantly related to their subsequent choice to self-administer a low or high dose of heroin (measured using a progressive ratio task), relative to healthy controls (80). Another study evaluated D2 receptor availability with and without naloxone administration using PET imaging in people with current DSM-IV opioid dependence and ongoing heroin use (*n* = 11) and controls without this opioid use history (*n* = 11) (81). Persons with opioid dependence showed decreased D2 receptor availability in the striatum compared to controls at presentation to the study. Precipitation of acute withdrawal using the opioid antagonist naloxone was not found to further decrease D2 receptor availability relative to control subjects, though a *post-hoc* analysis did suggest that persons with opioid dependence who received higher naloxone doses (0.02 mg/kg; *N* = 7) demonstrated greater reductions in D2 relative to persons who received a lower naloxone dose (0.01 mg/kg; *N* = 2).

### The D2 Family of Receptors May Become Supersensitive Once Opioid Physical Dependence Develops

The evidence described above identifies a potential role for the D2 receptor family in the expression of opioid effects and introduces the notion that receptor quantity is not necessarily the only mechanism through which this occurs. This notion is supported by an abundance of data from animal studies that suggest chronic exposure to opioids leads to functional adaptations in the dopamine system that sensitizes the system to D2 agonists rather than changes in the quantity of receptors. This supersensitivity may, in turn, increase drug seeking by potentiating behavioral responses to D2-like activation or, theoretically, increase the reinforcing effects of D2 agonists. Consistent with changes observed over time in levels of striatal dopamine, supersensitivity also appears to last well-beyond the somatic resolution of withdrawal symptoms, suggesting they are enduring conformational changes.

For instance, doses of the D2 agonist quinpirole that are so low they produce no effect in control rats were shown to increase behaviors that resemble opioid withdrawal as well as stimulant-induced stereotypies in rats that were made dependent and then withdrawn from opioids. Moreover, quinpirole in that study was also shown to increase the rate of dopamine metabolism, an effect that was more pronounced at 48 than 24 h (82). Another study that administered the D2 receptor agonists propylnorapomorphine and quinpirole found they selectively increased locomotor activity in rats only once they had developed opioid physical dependence and were in a state of naltrexone-precipitated or spontaneous withdrawal; those effects were not observed when morphine was acutely administered to non-dependent animals or when the probe was a D1 receptor agonist (83). A comparison of the dopamine agonists apomorphine and dopamine to acetylcholine and prostaglandin E found that rats undergoing naloxone-precipitated withdrawal exhibited increased jumping behavior when apomorphine or dopamine were administered but showed no effect to the other substances; changes in jumping were also not evidence in animals that were not physically dependent on opioids or in animals that were physically dependent but not in a state of withdrawal (84).

Examination with the D2 receptor agonist bromocriptine has revealed similar outcomes. In rats trained to respond for cocaine and heroin, bromocriptine was found to be more potent in reinstating responding for heroin than it was for cocaine, evidenced by its ability to reinstate heroin responding at lower dose ranges than for cocaine. Bromocriptine also revealed a time x dose interaction in which larger doses engendered substantially more responding when administered at later vs. earlier time points; this effect was only observed in the heroin-trained animals and the cocaine-trained animals showed no such effect (85). An examination of dopamine sensitivity and receptor quantity provides further evidence that these effects are not a byproduct of D2 receptor upregulation. Specifically, administration of the D2-probe [3H] spiroperdiol in rats that were chronically exposed to opioids revealed no differences in the number of D2-receptor binding sites, regardless of whether the rats were receiving opioid agonists, in a state of withdrawal, or were opioid-naïve control animals. In contrast, administration of the selective D2 agonist bromocriptine to animals that had opioid physical dependence increased their locomotor and stereotypic responses relative to control animals (86). Finally, a comparison of morphine and amphetamine in dogs found that initial doses of morphine increased locomotive behavior but did not produce the same type of stereotypies observed following amphetamine exposure; however, after repeated small doses of morphine stereotypies emerged, suggesting a supersensitivity had developed in response to repeated opioid agonist administration (87).

This effect has been rarely studied in humans and it is difficult to know whether the decreased D2 levels reported by the PET studies above reflect acute changes in D2 as a function of chronic opioid exposure (which would suggest it is the mechanism through which opioids may influence stimulant co-use) or whether reduced D2 levels precede the acquisition of opioid misuse. This latter point is supported by several studies that have implicated reduced D2 receptor density as a predictor of the strength of the reinforcing effects of drugs that exert dopaminergic activity (88–90). We know of only one study that examined dopamine supersensitivity in persons as a function of opioid exposure. That study conducted a venotest wherein small test doses of serotonin and dopamine were administered to men (*n* = 7) who had opioid physical dependence to measure changes in their smooth muscle response using orthodromic incanulization. When tested 3–12 h after their last opioid exposure, exposure to small challenge doses of serotonin and dopamine resulted in 100 and 1,000-fold changes in venous pressure, respectively. In contrast, norepinephrine had no effect. The participant with the most proximal exposure to heroin (3 h prior) showed the strongest response to dopamine, a 1,000-fold change. Naloxone administration reversed the direction of effects and decreased levels by 100 and 1,000-fold, and re-administration of morphine was able to restabilize levels (demonstrating causal relationships) (91). These data support the preclinical data presented and indicate that supersensitivity may at least play a role in the human experience.

### Supersensitivity of the Dopamine System Continues to Intensify as the Opioid Withdrawal Syndrome Transitions From Acute to Protracted

Several studies that have examined the time course of dopamine supersensitivity have found that mild supersensitivity is evident almost immediately after the last opioid exposure in physically dependent animals and that supersensitivity continues to increase in strength over time, such that sensitivity peaks several days after the final opioid exposure. For instance, during a period of spontaneous opioid withdrawal, rats trained to nose-poke for heroin that received the D2 receptor family agonist quinpirole emitted a sensitized locomotor response around day 4 of withdrawal (with effects resolving by 21-days) (92). This outcome was also observed in rats that were withdrawn from morphine and followed over an 8-week protracted withdrawal period. These animals exhibited relatively low rates of lever pressing in response to morphine during the protracted withdrawal period but increased responding for the D2 agonist apomorphine. Moreover, the ability of apomorphine to elicit responding increased during the protracted period relative to when rats were physically dependent on opioids (93). Microdialysis studies have also found that although extracellular dopamine levels are increased by 35% in response to a morphine dose in animals that have been withdrawn from opioids for 2 days, administering morphine on days 3 and 5 of opioid abstinence increased dopamine levels by as much as 160% and this potentiation of dopamine release only began to resolve by day 7 of abstinence (the final day evaluated in this study) (76).

Another method for evaluating dopamine supersensitivity is through unilateral lesioning of dopamine neurons either through electrolysis or administration of the 6-OHDA dopamine-neurotoxin. In animals that have received a unilateral striatal lesion, dopaminergic agonism and antagonism produces ipsilateral and contralateral turning behaviors, respectively (94). Evidence suggests that rats with 6-OHDA lesions will elicit ipsilateral turning behavior in response to opioid agonists but not antagonists and that this behavior can be blocked by naloxone; these data support the notion that opioids confer dopaminergic effects and suggest this assay is useful for detecting opioid-induced changes in behavior (72, 95, 96). Consistent with the aforementioned evidence, 6-OHDA-related turning behavior is not evident when a single acute opioid dose is examined; it only emerges following chronic opioid exposure and then increases in frequency as opioid tolerance develops (96). In addition, once animals have developed a physical dependence on opioids, naloxone administration produces contralateral (e.g., antagonistic) turning behavior (72) which can be reversed by provision of the stimulant D2 agonists apomorphine and d-amphetamine (95). Co-administration of apomorphine and morphine in non-tolerant rats has also been found to increase ipsilateral (e.g., agonist) circling behaviors in an additive manner, signifying a dopamine agonist effect. Moreover, once an animal that has developed opioid physical dependence has been withdrawn from opioids, morphine will no longer elicit a turning response; however, apomorphine will continue to elicit the ipsilateral (e.g., agonist) turning response in animals during a period of withdrawal, and the intensity of the turning behavior has been found to increase as a function of time since last opioid exposure (96).

Finally, a series of behavioral assays provide additional evidence that the D2 receptor family becomes sensitized with extended opioid exposure. One study found that rats that were withdrawn from opioids exhibited excessive locomotor behavior on a rotometer during the withdrawal period that did not decrease to normal rates for 2-months (97). This effect has also been examined using aggression as a behavior metric of dopaminergic activity and supersensitivity. One such study found that rats that received d-amphetamine while undergoing spontaneous opioid withdrawal exhibited pronounced enhancement of aggression that was evident immediately and increased in severity when d-amphetamine was administered at various points during the 70-h post-withdrawal observation period (98). A second study that withdrew rats from morphine and followed them for a 30-day period found that aggressive behaviors that were observed during opioid withdrawal could be blocked entirely by lesioning the nigrostriatal bundle (demonstrating a causal effect of the dopamine system in this behavior) and restored in lesioned animals through administration of the D2 receptor agonist apomorphine. Moreover, the dopamine turnover rate in the rats undergoing withdrawal, a measure of dopamine sensitivity, was also not found to differ between control and opioid-dependent animals prior to withdrawal but was significantly reduced in animals that had been withdrawn from opioids at a 30-days observation (99).

## What Human Evidence Do We Have?

The preponderance of evidence for hypotheses concerning dopamine supersensitivity has been generated in preclinical studies; only a limited number of human studies are able to contribute to this discussion and none of them were prospectively designed to evaluate these specific hypotheses. Thus, the data presented below, comprised of correlational, retrospective, or secondary analyses, should be considered as preliminary evidence to support more focused research. Nevertheless, we present them here to provide some evidence that the dopamine system is both integral to opioid-based effects and becomes disrupted following extended opioid exposure and/or abstinence in humans.

### Evidence That Opioids Produce Stimulating Effects in Humans

Only a few studies have examined the role of the dopamine system in the opioid physical dependence syndrome in humans. However, these studies do provide some preliminary evidence that corroborate the reviewed preclinical data by suggesting that supersensitivity to dopaminergic effects can be observed in humans following chronic opioid exposure as well as during periods of opioid abstinence. Two companion studies retrospectively assessed the experience of opioids in populations of individuals who were exposed to opioids for pain management and either did or did not continue on to develop opioid misuse or OUD. The first found that the initial subjective experience of opioids in persons who developed misuse behaviors (*n* = 20) was remembered as producing more opioid and stimulant-like effects, as determined by Addiction Research Center Inventory (ARCI) ratings, than was experienced by persons who did not continue on to develop misuse behaviors (*n* = 20) (100). A subsequent retrospective study by this group replicated the same ratings on the ARCI in a larger sample, and also found that persons who ultimately developed OUD (*n* = 39) were more likely to remember their first experience as producing effects consistent with increased dopaminergic activity, including feeling happy and experiencing greater activation than did persons who did not develop OUD (*n* = 40) (101). This effect has also been reported in laboratory studies. The first was a within-subject laboratory study that administered ascending doses of d-amphetamine and hydromorphone to individuals who had a history of opioid and stimulant co-use (*n* = 5) who then rated their subjective experience on the ARCI. The two highest doses of d-amphetamine administered (15 mg, 30 mg) produced scores on the morphine scale of the ARCI that exceeded the level produced by highest dose of hydromorphone (12 mg); in addition, 8 and 12 mg of hydromorphone produced a rating on the amphetamine scale consistent with 15 and 30 mg of d-amphetamine (102). The second was a within-subject human laboratory study that administered cocaine, hydromorphone, and cocaine/hydromorphone to persons with a history of cocaine and opioid use (*n* = 8). This study reported that cocaine (20, 40 mg) produced higher ratings on the morphine ARCI scale than did hydromorphone (1.5 mg, 3.0 mg) and that hydromorphone 3.0 mg produced higher ratings than cocaine on the ARCI amphetamine scale (103). Collectively these data provide evidence that opioids can produce a stimulating effect in humans, consistent with the preclinical work cited in the section above.

### Evidence That Individuals With Opioid Physical Dependence Experience Positive Effects From Stimulants

The limited number of studies that have investigated the experience of stimulants in persons who have opioid dependence collectively suggest stimulants confer unique effects in that population. Several of these studies have been conducted in the context of the emergent twin epidemic of opioids and methamphetamine co-use and present qualitative descriptions of rationales for this co-use from people with lived experience. The first collected semi-structured interviews from people in Appalachian Kentucky who had a history of non-medical opioid and methamphetamine use (104). That study identified key person-level motives to use that include: (1) suppressing withdrawal and craving for opioids, (2) achieving an attractive or desirable high, and (3) addressing underlying mental or physical health needs. These motives are not selective to this population; similar themes have been consistently observed across demographically and geographically diverse groups of people such as people who inject drugs or use opioids in rural Oregon (105) and those entering treatment across admission sites in the United States (4) and more globally (106).

Additional studies provide more concrete evidence that the dopamine system is activated during opioid withdrawal in humans. The first was a human laboratory study that evaluated naloxone-precipitated opioid withdrawal in persons with opioid physical dependence that did (*n* = 19) or did not (*n* = 33) also report using cocaine (107). Withdrawal severity was observed to be lower in patients who had concurrent cocaine use relative to those who had exclusive opioid use across the full-time course examined. An accompanying preclinical experiment in that paper reported that acute cocaine (20 mg/kg) was also able to reduce the severity of naloxone-precipitated withdrawal in rats. However, these data contrast with a survey study wherein people (*n* = 89) who had opioid physical dependence indicated that stimulating drugs (cocaine, amphetamine, nicotine, caffeine) were perceived as being less useful than depressants (e.g., benzodiazepines and alcohol) or cannabis at treating their opioid withdrawal. The majority of those patients felt that cocaine (62% of patients) and amphetamine (62%) increased the severity of the withdrawal syndrome, the highest for all drugs queried (108). This conflicting evidence may relate to the period when these stimulant drugs are administered (e.g., early or preempting withdrawal vs. during peak withdrawal period), duration of opioid use, or the stimulant dose administered; more systematic work is needed to evaluate these possibilities.

A third study used data from a 24-week randomized clinical trial comparing participants (*n* = 125) who were randomly assigned to varying doses of methadone (35 or 65 mg) or buprenorphine (2 or 6 mg buprenorphine) and found that subjects who received low doses of methadone or buprenorphine reported lower withdrawal in weeks wherein they had co-occurring cocaine use vs. weeks where they did not have co-occurring cocaine use (109). In contrast, patients who received high doses of buprenorphine reported higher withdrawal in weeks with co-occurring cocaine use. A dual model was proposed in which high maintenance doses of opioid drugs may result in a sensitivity to stimulant-induced withdrawal expression, a hypothesis consistent with some of the preclinical literature reviewed above, whereas low dose maintenance may result in a context where stimulant drugs alleviate low-level persistent withdrawal symptoms.

### Evidence of Dopamine Supersensitivity in Humans With Opioid Physical Dependence

The small number of studies that have evaluated outcomes related to dopamine supersensitivity in persons with OUD can provide some evidence of this effect. Here we conceptualize reports of a desirable subjective high following stimulant administration to be suggestive of an increased sensitivity to the effects of dopaminergic compounds following a period of chronic opioid exposure and during acute (and possibly prolonged) abstinence. The first was a double-blind study that compared the subjective effects of intravenous cocaine (0, 12.5, 25, and 50 mg) in patients receiving methadone treatment (50 mg/day) to persons who had a history of non-medical opioid use without any current opioid physical dependence. In that study cocaine was observed to produce greater positive subjective effects (e.g., good effect, like drug) for participants maintained on methadone compared to those who did not have opioid physical dependence (110). A second double-blind, human laboratory study administered varying doses of intravenous cocaine (0, 8, 16, 32, and 48 mg/70 kg) to patients maintained on methadone. Patients maintained on the highest dose range of methadone (90–100 mg) showed greater ratings of positive subjective effects to acute cocaine administration compared to those maintained on lower dose ranges, although these findings were limited by the small sample (*n* = 16) and lack of randomization to methadone dose (111). In contrast to these studies however, a third study reported no effect of buprenorphine maintenance on subjective effects produced by intravenous cocaine (30 mg) using a within-subject pre (before maintenance) post (after maintenance) design (112). It is possible that differences in the intrinsic efficacy between methadone and buprenorphine contributed to this discrepancy or that participants had already achieved high levels of opioid exposure resulting in a ceiling effect.

## Hypothesized Neural Circuit of Dopamine D2 Hypersensitivity During Opioid Withdrawal

Above, we described in detail preclinical and clinical data which suggests that D2 receptor hypersensitivity occurs specifically following opioid dependence and during states of acute or protracted opioid withdrawal, and that this change deviates from what is understood about stimulants alone and appears unique to stimulants in the context of opioid physical dependence. It is critical to understand how neural circuit changes due to chronic opioid use may differ from those that have been defined following use of chronic use of stimulants, which may also explain the emergence of psychomotor stimulant use among persons with OUD without premorbid chronic stimulant use. Preclinically, several studies have shown that withdrawal from cocaine induces a D1-driven mechanism, which drives cocaine seeking via disinhibition of the dopaminergic ventral mesencephalon, which in turn disinhibits the thalamus (113). Previously, it was thought that D1- and D2-expressing MSNs uniquely define the “direct” and “indirect” pathways projecting out of the striatum, originally from the dorsal striatum (114) and then later applied to the ventral striatum in the context of reward learning and cocaine use [e.g., (37, 115)]. However, more recent evidence suggests this dichotomy is inaccurate (36) as both D1- and D2-expressing MSNs project to the striatomesencephalic pathway and the striatopallidal pathway (116). Notably, in some of this work, none of the D2 MSNs identified appeared to project to the ventral mesencephalon (116). Moreover, another study found neurons projecting from the nucleus accumbens to dopamine neurons within the VTA that were inhibited by dopamine acting on D2 receptors (51). Collectively, these data indicate it is possible for a subpopulation of D2-expressing MSNs to project directly from the nucleus accumbens to the VTA. Despite the desegregation of D1 and D2 from the “direct” and “indirect” pathways, it has been repeatedly shown that D1-expressing MSNs are critical in driving cocaine seeking behavior (117–119), with a potential impairment in D2 inputs to the ventral pallidum to promote D1-driven cocaine seeking (120).

**Figure 2:**
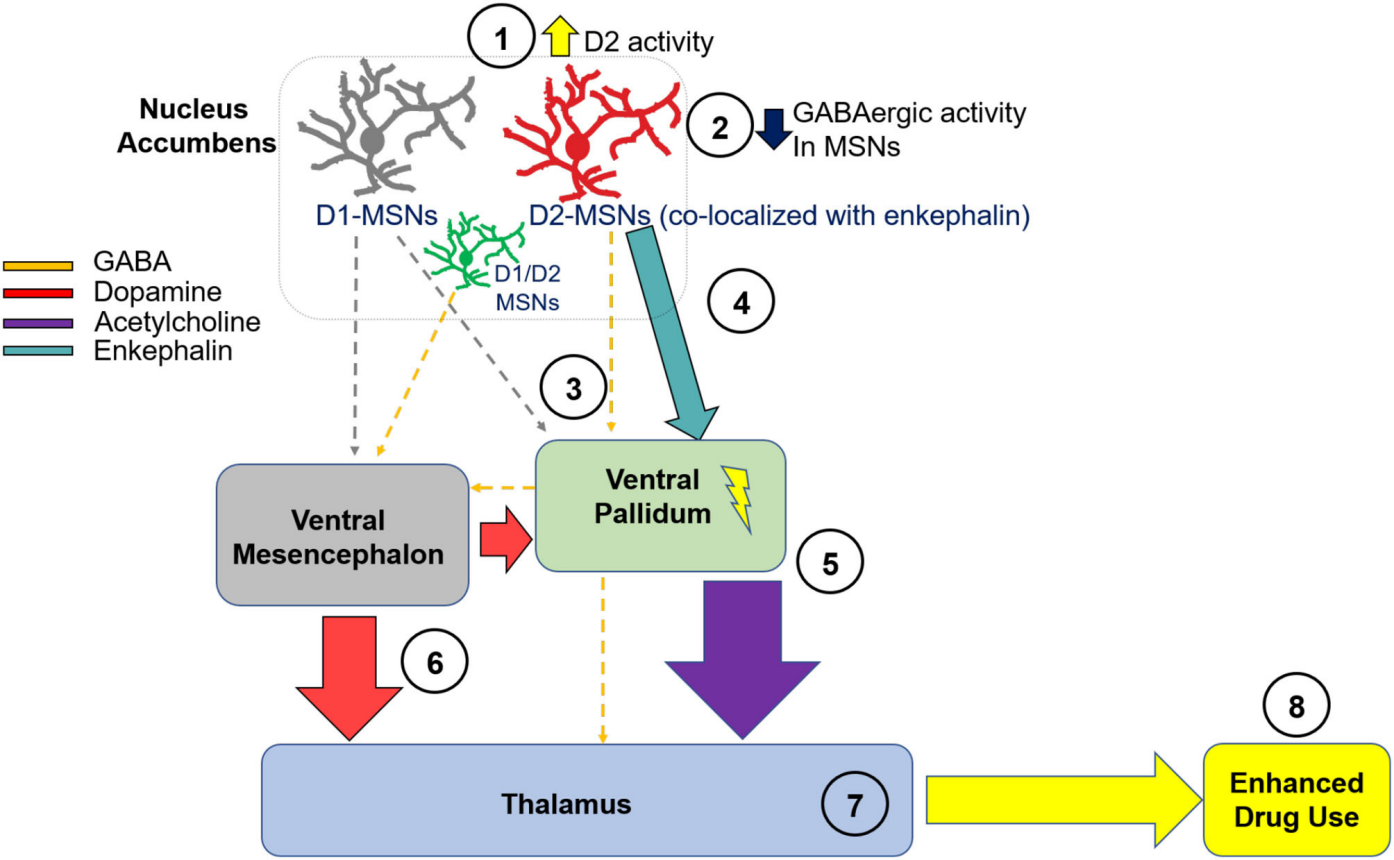
Hypothesized Neural Circuit of D2 Hypersensitivity in Opioid Withdrawal. We hypothesize that D2 receptor activity is during withdrawal from chronic opioid use (1), which leads to decreased activity of GABAergic MSNs within the NA (2). This leads to a reduction in GABAergic inhibitory tone from the NA to the VP (3). Through the direct projection to the VP, disinhibition of the VP from reduced NA-derived GABAergic innervation leads to enhanced excitability of cells residing in the VP. It is also possible that MSNs co-expressing D1 and D2 receptors project from the NA to the VM, and play a critical role in enhancing dopaminergic signaling from the VM to output structures. As well, enhanced enkephalin activity may enhance neuronal excitability within the VP (4). This excitation leads to enhanced acetylcholine release into the MD (5), which may enhance reward. Through the direct GABAergic projection from the VP to the mesencephalon, there is less inhibitory tone and consequently, enhanced dopaminergic activity in cells residing in the VM and projecting back to the VP or to the MD (6). The net result of these neural adaptations is enhanced excitability of thalamic nuclei including the MD (7), and, consequently, enhanced use of dopamine agonists during opioid withdrawal (8). NA, nucleus accumbens; VP, ventral pallidum; VM, ventral mesencephalon; MD, mediodorsal thalamic nucleus; D2, dopamine receptor D2; D1, dopamine receptor D1; MSN, medium spiny neuron.

We now propose a novel neural circuit mechanism through these pathways, one that is uniquely consequential to chronic opioid use and withdrawal. It should be noted that the entirety of this circuit is based on hypotheses derived from neuroanatomical literature, and each of the steps within the proposed pathway need to be empirically tested. Although stimulants may strengthen D1 innervation of terminal fields, we hypothesize that it is through strengthening of D2s that opioid withdrawal enhances the reinforcing effects of dopamine agonists, as well as alter other behaviors such as locomotor activity as described above. This hypothesis is supported by the fact that D1 agonists do not appear to have enhanced locomotor activity or show greater reinforcing efficacy following withdrawal from opioid use as well-one study finding that D2 receptors can suppress lateral inhibition from indirect MSNs to direct MSNs, which enhances the D1 output pathway in cocaine's stimulant actions [although, this suppression was specific to the collateral transmission, and did not impact transmission to the ventral pallidum; (121)]. This study specifically examined mechanisms relevant to cocaine, and it is not clear if collateral transmission would be enhanced or decreased following opioid use. Thus, in our hypothesized circuit (Figure 2), we have grayed the D1 projections from the nucleus accumbens to the ventral mesencephalon and the ventral pallidum. However, we acknowledge that this pathway may play a critical role in dopamine disinhibition in output structures, and thus we have included dopamine input from the ventral mesencephalon into the ventral pallidum and thalamus. Here we will systematically describe a potential novel circuit which we derived both from the relevant opioid and cocaine literature, and from a large body of neuroanatomical literature that has defined neurocircuitry in detail.

In Figure 2, we show a complex multi-step circuit, beginning in the nucleus accumbens (there are numerous glutamatergic projections into the nucleus accumbens as well, which we acknowledge may play a role in modulating nucleus accumbens circuit activity but are not included here). We propose that D2 receptors expressed on accumbens MSNs (122) originating in the nucleus accumbens and projecting to the ventral pallidum show enhanced functional activity (1). Given that D2 receptors are G_i/o_ coupled inhibitory receptors (123, 124), they function as autoreceptors (32) and their activation would reduce GABAergic tone into terminal regions. Thus, hypersensitivity of D2 receptors located on accumbens MSNs would result in inhibition of GABAergic MSNs (2) projecting to the ventral pallidum (3) (113, 116). Importantly, it has been previously shown that inhibition of ventral striatal terminals into the ventral pallidum via upregulation of D2 receptors in the nucleus accumbens enhances motivation (125), thus supporting this potential mechanism in the proposed circuit. Importantly, ventral striatal projections from the nucleus accumbens to the ventral pallidum include cells the express mRNA of both glutamate decarboxylase [GAD; a rate-limiting enzyme that catalyzes the conversion of glutamate to GABA and is thus used as a marker for GABA-containing cells; (126)], and the peptide enkephalin (127, 128), which comprise 46% of projecting neurons (129). Although it is unclear if there are enkephalin-containing neurons that do not co-express D2, there are studies showing that a third neuronal subtype exists which contain both D1 and D2 mRNA (130, 131), and which express D1-D2 heteromers (132, 133). Although unknown, it is possible that D2 hyperactivity through this subset of neurons disinhibits enkephalin input into the ventral pallidum. This is premised on prior data showing that enkephalin indirectly exerts excitatory tone on hippocampal pyramidal cells via blockade of spontaneous and evoked inhibitory potentials, and inhibitory pathways are depressed by enkephalin (134). Thus, the ventral pallidum may be disinhibited by cells projecting from the nucleus accumbens via enkephalin (4). It is also possible that D1/D2 co-expressing MSNs comprise a third subpopulation of cells, which project GABAergically to the ventral mesencephalon (51). These neurons may play a critical role in opioid withdrawal and enhancement of dopamine sensitivity following chronic opioid use, because it has been previously shown that disinhibition of dopamine neurons induced by chronic opioid use involves multiple GABA inputs, and these pathways are selectively sensitive to μ opioid receptor agonists (51).

The next step in this circuit involves ventral pallidum projections to the mediodorsal thalamic nucleus, which is a primary terminal region of the ventral pallidum (135). Although this early study unsuccessfully determined the neurotransmitter system(s) of the ventral pallidum-mediodorsal thalamic nucleus projection, later studies determined that this projection contains both GABAergic (GAD-positive; 53%) and cholinergic (ChAT-positive; ~16%) neurotransmitters (136, 137). Importantly, one prior study showed that both feeding and d-amphetamine administration enhanced extracellular acetylcholine in the medial thalamus, identifying a possible role of acetylcholine in this region in reward (138). Thus, although a much smaller proportion of cells as compared to GABA, it is possible that activation of the ventral pallidum may enhance cholinergic input into the mediodorsal thalamic nucleus, thus driving drug use during opioid withdrawal (5). Next, we describe potential dopaminergic modulation of the thalamus in our hypothesized circuit. Given that the ventral pallidum sends GABAergic projections to the dopaminergic mesencephalon (128, 139), we hypothesize that this may be disinhibited as a consequence of chronic opioid use, leading to enhanced dopaminergic tone into output structures of the mesencephalon including a loop back to the ventral pallidum and also to the thalamus, as it has been previously shown that dopaminergic neurons of the ventral mesencephalon project bilaterally to the thalamus (140). As well, it is possible that D1-MSNs do not send strong GABAergic tone into the ventral mesencephalon after chronic opioid use, given that D1 receptors do not appear to be involved in hypersensitivity to dopamine agonists.

The net result may be enhanced dopaminergic signaling due to dopamine agonists during withdrawal from chronic opioids. It has been previously established that the ventral pallidum receives dopaminergic innervation from the ventral mesencephalon (141). Because the mesencephalon contains a mix of A9 and A10 midbrain dopamine neuron subtypes (142, 143), we hypothesize that this group of midbrain structures projects dopaminergically into the thalamus (6) and enhances its activity following opioid use (7). We also hypothesize that this projection, along with the accumbens-pallidal-thalamic projection [steps 1–5], plays a potential role in driving enhanced sensitivity to dopamine agonists during opioid withdrawal (8). Recently, there has been an interest in the role of thalamic nuclei in addiction (144), and thus we hypothesize that this is a critical output structure involved in opioid withdrawal-induced enhancement of dopamine agonists.

## How Can These Data Inform Translational Research

Collectively, these data suggest that following chronic exposure to an opioid and development of opioid physical dependence, the dopamine system appears to operate in a typical manner when an opioid agonist is concurrently present. However, the absence of an opioid agonist causes a disruption of dopaminergic signaling that is evident very shortly after the final opioid exposure occurs, and that disruption grows in severity and intensity as the acute withdrawal period extends into the protracted withdrawal period. Studies that examined long-term changes in functioning suggest that alterations in dopaminergic signaling may not resolve for several weeks. Although some data have been collected in human laboratory and clinical settings that may inform this hypothesis, the specific degree to which dopamine supersensitivity intensity occurs and the time course over which it develops and resolves in humans is uncertain. Moreover, differences in how opioid withdrawal is expressed, as well as its normal time course, between animals and humans makes it challenging to directly translate the preclinical evidence to the human clinical condition. Nevertheless, a few noteworthy conclusions from this review can be made, each of which point toward critical translational steps for future research with broader implications for the stimulant-opioid co-use epidemic as well as opioid relapse (see also Table 1):

(1) Changes in the dopamine system occur only once physical dependence to the opioid develops and the chronicity of opioid exposure is associated with the magnitude of changes.
(2) Opioid withdrawal leaves the organism in a state of substantive dopamine deficit.
(3) Changes in dopamine levels and signaling persist long after the somatic or observed signs of opioid withdrawal appear to have resolved (thus, organisms that appear to have resolved the acute withdrawal syndrome may be continuing to function in a dysregulated state, suggesting continued sensitivity to acute withdrawal consequences).
(4) Once physical dependence occurs, a state of dopamine supersensitivity develops very soon after abstinence from opioids begins.
(5) Supersensitivity to drugs that function as dopamine agonists (including low doses of opioids and otherwise subthreshold doses of dopamine agonists) increases as the abstinence period continues and is evident several weeks into the protracted withdrawal period.
(6) Changes appear to be driven by conformational changes in the sensitivity but not quantity of the D2-family of receptors.

**Table 1:** Notable conclusions, clinical implications, and future research directions.

**Notable conclusions**
1	Changes in the dopamine system occur only once physical dependence to the opioid develops and the chronicity of opioid exposure is associated with the magnitude of changes
2	Opioid withdrawal leaves the organism in a state of substantive dopamine deficit
3	Changes in dopamine levels and signaling persist long after the somatic or observed signs of opioid withdrawal appear to have resolved (thus, organisms that appear to have resolved the acute withdrawal syndrome may be continuing to function in a dysregulated state, suggesting continued sensitivity to acute withdrawal consequences)
4	Once physical dependence occurs, a state of dopamine supersensitivity develops very soon after abstinence from opioids begins
5	Supersensitivity to drugs that function as dopamine agonists (including low doses of opioids and otherwise subthreshold doses of dopamine agonists) increases as the abstinence period continues and is evident several weeks into the protracted withdrawal period
6	Changes appear to be driven by conformational changes in the sensitivity but not quantity of the D2-family of receptors
**Clinical implications**
1	Stimulant-opioid co-use may confer euphoric effects that are greater than what is produced by either drug alone or what may be experienced by persons who do not have opioid physical dependence
2	Stimulants may partially remediate symptoms of opioid acute withdrawal, thus reinforcing stimulant-opioid co-use
3	Opioid acute and protracted withdrawal may be characterized by a hypo-dopaminergic state during which an individual may experience an enhanced motivation to restore dopamine function that can manifest as craving and/or opioid relapse
**Future research directions**
1	Evaluate presence and time course of dopamine supersensitivity in humans with opioid physical dependence during periods of opioid maintenance and withdrawal
2	Evaluate new and/or repurposed D2 agonists or antagonists for stimulant-opioid co-use treatment, opioid withdrawal remediation, and/or opioid relapse prevention/craving remediation

### Implications for Increased Reinforcing Effects of Stimulants

#### Stimulant-Opioid Co-use for Euphoric Effects

Supersensitivity of the dopamine system that develops following chronic opioid exposure would presumably increase the reinforcing effects of dopaminergic agonists (such as cocaine and methamphetamine) beyond what might be experienced in people who are using opioids but have not yet developed opioid physical dependence and at levels that could possibly be greater than what is experienced in non-tolerant, opioid-naïve individuals. If true, this hypothesis would suggest that exposure to a stimulant during a state of opioid physical dependence would produce a unique and robust reinforcing effect, which theoretically could increase the likelihood the drugs would be co-used.

Preclinical evidence already partly supports this suggestion. One experiment evaluating cocaine and the opioid agonist remifentanil in rodents showed increased sensitivity to cocaine (i.e., increased hedonic setpoints and reduced sensitivity to increasing response cost) among animals that had a greater prior exposure to the opioid remifentanil (145). This effect was not reciprocal; prior exposure to cocaine was not associated with later remifentanil use motivation. These data suggest that exposure to opioids prior to cocaine administration increased cocaine reinforcement in a manner that was directionally and pharmacologically-specific. Another study found that among non-human primates, motivation to use cocaine was higher during periods of morphine withdrawal and that this period of increased use extended four-to-five weeks after chronic opioid exposure ended (146). The human laboratory data reviewed above similarly partly support this notion, for example, with greater subjective effects of intravenous cocaine observed among those with a history of opioid physical dependence (110). Systematic and controlled studies to this end are needed.

#### Stimulant Use for Opioid Withdrawal Remediation

Another pathway through which co-use could be reinforced is by remediation of the acute opioid withdrawal syndrome. The daily pattern of opioid use is generally characterized by frequent administration of a short-acting opioid several times a day. Functionally, this means that during the inter-dose interval an individual will start moving into a state of acute opioid withdrawal several times throughout the day. The data reviewed here suggest acute withdrawal is associated with both a dopamine depletion and development of dopamine receptor supersensitivity that can emerge following even a short period of opioid abstinence and whose magnitude is at least somewhat related to the chronicity of prior opioid exposure. Thus, exposure to a dopamine agonist during a period of transition into acute withdrawal could theoretically produce a reinforcing effect that is enhanced relative to its administration in a non-opioid dependent state, and which might engender additional co-use behavior. Although these effects have not been systematically evaluated in humans, the data reviewed here reveal a putative mechanism through which dopaminergic agonists could produce extra-stimulating effects and some evidence for mitigation of this withdrawal syndrome that might strongly maintain co-use behavior. However, evidence also suggest a possibility for precipitation of opioid withdrawal-like symptoms following stimulant administration among a subset of patients. These findings emphasize the need for parametric evaluation of factors that impact the precipitation vs. alleviation of opioid withdrawal by stimulants drugs to include history of use, timing of administration, and type of dopamine agonist.

### Implications for Opioid Relapse

In clinical practice, the period of time after an individual is fully withdrawn from opioids is characterized by excessively high rates of opioid relapse, particularly during the first 30 days. Relapse during this period is also extremely dangerous; the lack of opioid tolerance following withdrawal raises the risk of fatality due to overdose to a level higher than at any other point during a person's opioid use history. It is recognized that people who have been withdrawn from opioids experience a protracted withdrawal syndrome, and while the actual composition of that syndrome has not been sensitively characterized it is generally believed to consist of persistent mood disruptions, craving, and sleep disturbance. The clinical importance of the protracted withdrawal symptoms is often overshadowed by the more visible and better characterized acute withdrawal syndrome, around which most of our opioid-related treatments are organized.

The data reviewed here provide evidence that the resolution of observable and/or somatic withdrawal symptoms does not reflect a resolution of the acute withdrawal syndrome and that the organism is likely still in a state of dopamine deficit even once overt signs of physical withdrawal symptoms have abetted. Dopamine deficits have themselves been independently associated with mood impairments, suggesting this state could be responsible for some of the mood-related symptoms generally characterized as protracted withdrawal. Moreover, the fact that dopamine signaling is not only dysregulated, but may become super-sensitized during the immediate protracted period, provides a putative mechanism through which the excessively high rates of relapse to opioids in early abstinence may occur. Specifically, the collective data reviewed suggest that during a state of dopamine supersensitivity, exposure to a drug that produces a stimulating effect (a low dose of an opioid or of a stimulant) may produce a more robust and reinforcing effect than it would have produced during a state of opioid satiety (prior to withdrawal). Data further suggest that this effect will become stronger over time before eventually stabilizing several weeks later. Although hypothetical, this supersensitivity could manifest to the individual as a general “urge” or “craving” to use a substance, particularly something that they have previously associated with the restoration of dopamine levels (147). This is supported by evidence that craving for opioids also increases in severity following withdrawal from opioids (148), a phenomenon referred to in the preclinical field as “incubation of craving” (149). It is therefore plausible that the dopamine deficit and resultant supersensitivity that is present following opioid withdrawal could be driving increases in opioid-related craving. In a state of dopamine deficit and supersensitivity, exposure to even low doses of opioid or stimulant could theoretically produce a reinforcing effect that is higher than what had been recently experienced and precipitate a relapse to regular opioid use.

## Competing Hypotheses

The collective data reviewed here support a novel and testable hypothesis that (if true) would advance our understanding of why stimulant and opioid co-use occurs, as well as inform risk for opioid relapse during periods of acute abstinence. As this hypothesis remains untested, it is important to acknowledge competing hypotheses that may also explain these same behaviors. One example is the Reward Deficiency Syndrome (RDS), which hypothesizes that chronic opioid exposure produces a hypodopaminergic state that leads to compulsive drug seeking [see (150–152) for review of RDS]. The reward deficiency syndrome posits that genetically-mediated (e.g., trait) differences between individuals underlie differential dopamine function and subsequent drug use behavior. Our hypothesis posits that the same individual could move in and out of a state of dopamine supersensitivity as their opioid physical dependence changes over time (e.g., state-based differences). It is therefore possible that these two theories could be operating in parallel. However, it is also possible for these theories to be competing with each other, and some of the data reviewed here support both potential theories. For instance, the clinical PET imaging data reviewed do not strongly support our current hypothesis, though they were also not designed to examine D2 supersensitivity and were conducted with small and selective samples (e.g., predominately male); thus, the degree to which they support or refute this theory is uncertain. We also did not uncover any preclinical studies that examined receptor function in the context we described, namely a period of acute abstinence from opioids in animals that had established opioid physical dependence. It is also possible that the effects we describe are driven by neuroadaptations in other non-dopamine substrates or circuits. The vast majority of studies reviewed in support of this hypothesis were conducted several decades ago and reported outcome measures that do not reflect current techniques or a contemporary understanding of neural architecture and function, so these questions remain untested.

## Conclusions and Call for Future Research

Collectively, this existing evidence base outlines putative mechanisms to understand how conformational changes to the dopamine system in persons with opioid physical dependence may contribute meaningfully to opioid-stimulant co-use as well as opioid-relapse behavior. This hypothesis is based almost exclusively on animal research models, which are highly rigorous but challenging to translate to the human condition. More research is needed in human models to examine dopamine supersensitivity following development of opioid physical dependence. These data also provide potential pathways for medication development. A variety of D2 receptor family medications exist on the market for other indications that could be repurposed as treatments for new onset stimulant use in persons with opioid use disorder and/or opioid relapse prevention or opioid withdrawal remediation. This may include a dopamine agonist replacement approach using D2 agonists such as bromocriptine, pergolide, lisuride, ropinirole, risperidone, and prampipexole or D2 partial agonists aripiprazole and brexpiprazole. Additional work may also focus on D2 receptor antagonism using medications such as buspirone, metoclopramide, tiapride, or raclopride. It is acknowledged that several prior attempts to utilize agonist replacement or D2-specific treatments for stimulant use disorder have been ineffective, and that several of these medications are also recognized as producing somewhat low or minimal effects for their indicated conditions (153, 154). However, since the data presented here indicate these medications may exert more potent effects in persons with opioid physical dependence than the general population and that these effects may be especially relevant during withdrawal from opioids, these approaches should not be ruled out on the basis of those prior studies. These data suggest that the population of people who have developed opioid physical dependence will likely have a unique response to dopaminergic medications. Importantly, the fact that these FDA-approved medications are largely unscheduled means that, if effective, there would be few barriers to their clinical adoption. Such an approach could help dramatically scale up treatment access and provide a method to combat the growing co-use epidemic, as well as provide an empirically-supported method to augment existing opioid treatment paradigms. In the context of an ever growing and evolving opioid crisis, with increasing morbidity and mortality, innovative approaches are needed, and the data reviewed here provide a pathway for exploration that is worth pursuing.

## Author Contributions

All authors conceptualized the topic, drafted the manuscript, and reviewed and approved the final version.

## Funding

Writing of this review was supported in part by NIDA grant R01DA052937 (KD), R21DA044479 (CG), R01DA046526 (CG), R21DA049130 (CG), R03DA045881 (CG), and R03DA054098 (JS).

## Conflict of Interest

In the past 3 years, KD has consulted for Canopy Corporation, Beckley-Canopy, and MindMed. The remaining authors declare that the research was conducted in the absence of any commercial or financial relationships that could be construed as a potential conflict of interest.

## Publisher's Note

All claims expressed in this article are solely those of the authors and do not necessarily represent those of their affiliated organizations, or those of the publisher, the editors and the reviewers. Any product that may be evaluated in this article, or claim that may be made by its manufacturer, is not guaranteed or endorsed by the publisher.

## References

[1] Jones CM Bekheet F Park JN Alexander GC. The evolving overdose epidemic: synthetic opioids and rising stimulant-related harms. Epidemiol Rev. (2020) 42:154–66. 10.1093/epirev/mxaa011 33511987 PMC9200066

[2] Fischer B O'Keefe-Markman C Lee AM Daldegan-Bueno D. 'Resurgent', 'twin' or 'silent' epidemic? A select data overview and observations on increasing psycho-stimulant use and harms in North America. Subst Abuse Treat Prev Policy. (2021) 16:e17. 10.1186/s13011-021-00350-5 33588896 PMC7883758

[3] Jones CM Underwood N Compton WM. Increases in methamphetamine use among heroin treatment admissions in the United States, 2008-17. Addiction. (2020) 115:347–53. 10.1111/add.14812 31503384 PMC6982538

[4] Ellis MS Kasper ZA Cicero TJ. Twin epidemics: the surging rise of methamphetamine use in chronic opioid users. Drug Alcohol Depend. (2018) 193:14–20. 10.1016/j.drugalcdep.2018.08.029 30326396

[5] Cicero TJ Ellis MS Kasper ZA. Polysubstance use: a broader understanding of substance use during the opioid crisis. Am J Public Health. (2020) 110:244–50. 10.2105/AJPH.2019.305412 31855487 PMC6951387

[6] Strickland JC Stoops WW Dunn KE Smith KE Havens JR. The continued rise of methamphetamine use among people who use heroin in the united states. Drug Alcohol Depend. (2021) 225:108750. 10.1016/j.drugalcdep.2021.108750 34052690 PMC8282713

[7] Strickland JC Havens JR Stoops WW. A nationally representative analysis of “twin epidemics”: rising rates of methamphetamine use among persons who use opioids. Drug Alcohol Depend. (2019) 204:107592. 10.1016/j.drugalcdep.2019.107592 31586804 PMC6884137

[8] Palamar JJ Han BH Keyes KM. Trends in characteristics of individuals who use methamphetamine in the United States, 2015-2018. Drug Alcohol Depend. (2020) 213:108089. 10.1016/j.drugalcdep.2020.108089 32531703 PMC7371537

[9] Gladden RM O'Donnell J Mattson CL Seth P. Changes in opioid-involved overdose deaths by opioid type and presence of benzodiazepines, cocaine, and methamphetamine - 25 States, July-December 2017 to January-June 2018. MMWR Morb Mortal Wkly Rep. (2019) 68:737–44. 10.15585/mmwr.mm6834a2 31465320 PMC6715260

[10] Lim JK Earlywine JJ Bagley SM Marshall BDL Hadland SE. Polysubstance involvement in opioid overdose deaths in adolescents and young adults, 1999-2018. JAMA Pediatr. (2021) 175:194–6. 10.1001/jamapediatrics.2020.5035 33226412 PMC7684514

[11] Roehler DR Olsen EO Mustaquim D Vivolo-Kantor AM. Suspected nonfatal drug-related overdoses among youth in the US: 2016-2019. Pediatrics. (2021) 147:e2020003491. 10.1542/peds.2020-003491 33288728 PMC9541269

[12] Cano M Huang Y. Overdose deaths involving psychostimulants with abuse potential, excluding cocaine: state-level differences and the role of opioids. Drug Alcohol Depend. (2021) 218:108384. 10.1016/j.drugalcdep.2020.108384 33158665

[13] Han B Cotto J Etz K Einstein EB Compton WM Volkow ND. Methamphetamine overdose deaths in the us by sex and race and ethnicity. JAMA Psychiatry. (2021) 78:564–7. 10.1001/jamapsychiatry.2020.4321 33471025 PMC8100861

[14] Howell BA Bart G Wang EA Winkelman TNA. Service involvement across multiple sectors among people who use opioids, methamphetamine, or both, United States-2015-2018. Med Care. (2021) 59:238–44. 10.1097/MLR.0000000000001460 33165146 PMC7878287

[15] Shearer RD Howell BA Bart G Winkelman TNA. Substance use patterns and health profiles among US adults who use opioids, methamphetamine, or both, 2015-2018. Drug Alcohol Depend. (2020) 214:108162. 10.1016/j.drugalcdep.2020.108162 32652380 PMC8147519

[16] Kosten TR Rounsaville BJ Kleber HD. Antecedents and consequences of cocaine abuse among opioid addicts. A 25-year follow-up. J Nerv Ment Dis. (1988) 176:176–81. 10.1097/00005053-198803000-00006 3343591

[17] Hartel DM Schoenbaum EE Selwyn PA Kline J Davenny K Klein RS. Heroin use during methadone maintenance treatment: the importance of methadone dose and cocaine use. Am J Public Health. (1995) 85:83–8. 10.2105/AJPH.85.1.83 7832267 PMC1615273

[18] Leri F Stewart J Tremblay A Bruneau J. Heroin and cocaine co-use in a group of injection drug users in Montréal. J Psychiatry Neurosci. (2004) 29:40–7. 14719049 PMC305269

[19] Torrens M San L Peri JM Olle JM. Cocaine abuse among heroin addicts in Spain. Drug Alcohol Depend. (1991) 27:29–34. 10.1016/0376-8716(91)90083-B 2029857

[20] Leri F Bruneau J Stewart J. Understanding polydrug use: review of heroin and cocaine co-use. Addiction. (2003) 98:7–22. 10.1046/j.1360-0443.2003.00236.x 12492751

[21] Chan B Freeman M Ayers C Korthuis PT Paynter R Kondo K. A systematic review and meta-analysis of medications for stimulant use disorders in patients with co-occurring opioid use disorders. Drug Alcohol Depend. (2020) 216:108193. 10.1016/j.drugalcdep.2020.108193 32861136 PMC8562993

[22] Condelli WS Fairbank JA Dennis ML Rachal JV. Cocaine use by clients in methadone programs: significance, scope, and behavioral interventions. J Subst Abuse Treat. (1991) 8:203–12. 10.1016/0740-5472(91)90040-H 1787544

[23] Gastberger S Baumgartner MR Soyka M Quednow BB Hulka LM Herdener M. Concomitant heroin and cocaine use among opioid-dependent patients during methadone, buprenorphine or morphine opioid agonist therapy. Eur Addict Res. (2019) 25:207–12. 10.1159/000500542 31067528

[24] Ball JC Ross A Jaffe JH. Cocaine and heroin use by methadone maintenance patients. NIDA Res Monogr. (1989) 95:328. 2640981

[25] Hanbury R Sturiano V Cohen M Stimmel B Aguillaume C. Cocaine use in persons on methadone maintenance. Adv Alcohol Subst Abuse. (1986) 6:97–106. 10.1300/J251v06n02_07 3604790

[26] Rawson RA McCann MJ Hasson AJ Ling W. Cocaine abuse among methadone maintenance patients: are there effective treatment strategies? J Psychoactive Drugs. (1994) 26:129–36. 10.1080/02791072.1994.10472260 7931857

[27] Silverman K Higgins ST Brooner RK Montoya ID Cone EJ Schuster CR. Sustained cocaine abstinence in methadone maintenance patients through voucher-based reinforcement therapy. Arch Gen Psychiatry. (1996) 53:409–15. 10.1001/archpsyc.1996.01830050045007 8624184

[28] Poling J Oliveto A Petry N Sofuoglu M Gonsai K Gonzalez G. Six-month trial of bupropion with contingency management for cocaine dependence in a methadone-maintained population. Arch Gen Psychiatry. (2006) 63:219–28. 10.1001/archpsyc.63.2.219 16461866

[29] Margolin A Kosten TR Avants SK Wilkins J Ling W Beckson M. A multicenter trial of bupropion for cocaine dependence in methadone-maintained patients. Drug Alcohol Depend. (1995) 40:125–31. 10.1016/0376-8716(95)01198-6 8745134

[30] Di Chiara G North RA. Neurobiology of opiate abuse. Trends Pharmacol Sci. (1992) 13:185–93. 10.1016/0165-6147(92)90062-B 1604711

[31] Koob GF. Neurobiology of opioid addiction: opponent process, hyperkatifeia, and negative reinforcement. Biol Psychiatry. (2020) 87:44–53. 10.1016/j.biopsych.2019.05.023 31400808

[32] Ford CP. The role of D2-autoreceptors in regulating dopamine neuron activity and transmission. Neuroscience. (2014) 282:13–22. 10.1016/j.neuroscience.2014.01.025 24463000 PMC4108583

[33] Di Chiara G Imperato A. Drugs abused by humans preferentially increase synaptic dopamine concentrations in the mesolimbic system of freely moving rats. Proc Natl Acad Sci USA. (1988) 85:5274–8. 10.1073/pnas.85.14.5274 2899326 PMC281732

[34] Wise RA Newton P Leeb K Burnette B Pocock D Justice JB Jr. Fluctuations in nucleus accumbens dopamine concentration during intravenous cocaine self-administration in rats. Psychopharmacology. (1995) 120:10–20. 10.1007/BF02246140 7480530

[35] Wise RA Leone P Rivest R Leeb K. Elevations of nucleus accumbens dopamine and DOPAC levels during intravenous heroin self-administration. Synapse. (1995) 21:140–8. 10.1002/syn.890210207 8584975

[36] Smith RJ Lobo MK Spencer S Kalivas PW. Cocaine-induced adaptations in D1 and D2 accumbens projection neurons (a dichotomy not necessarily synonymous with direct and indirect pathways). Curr Opin Neurobiol. (2013) 23:546–52. 10.1016/j.conb.2013.01.026 23428656 PMC3681928

[37] Bock R Shin JH Kaplan AR Dobi A Markey E Kramer PF. Strengthening the accumbal indirect pathway promotes resilience to compulsive cocaine use. Nat Neurosci. (2013) 16:632–8. 10.1038/nn.3369 23542690 PMC3637872

[38] Lobo MK Covington HE 3rd Chaudhury D Friedman AK Sun H Damez-Werno D. Cell type-specific loss of BDNF signaling mimics optogenetic control of cocaine reward. Science. (2010) 330:385–90. 10.1126/science.1188472 20947769 PMC3011229

[39] Scofield MD Heinsbroek JA Gipson CD Kupchik YM Spencer S Smith AC. The nucleus accumbens: mechanisms of addiction across drug classes reflect the importance of glutamate homeostasis. Pharmacol Rev. (2016) 68:816–71. 10.1124/pr.116.012484 27363441 PMC4931870

[40] Gerfen CR Surmeier DJ. Modulation of striatal projection systems by dopamine. Annu Rev Neurosci. (2011) 34:441–66. 10.1146/annurev-neuro-061010-113641 21469956 PMC3487690

[41] Nadjar A Brotchie JM Guigoni C Li Q Zhou SB Wang GJ. Phenotype of striatofugal medium spiny neurons in parkinsonian and dyskinetic nonhuman primates: a call for a reappraisal of the functional organization of the basal ganglia. J Neurosci. (2006) 26:8653–61. 10.1523/JNEUROSCI.2582-06.2006 16928853 PMC6674386

[42] Matamales M Bertran-Gonzalez J Salomon L Degos B Deniau JM Valjent E. Striatal medium-sized spiny neurons: identification by nuclear staining and study of neuronal subpopulations in BAC transgenic mice. PLoS ONE. (2009) 4:e4770. 10.1371/journal.pone.0004770 19274089 PMC2651623

[43] Chioma VC Kruyer A Bobadilla AC Angelis A Ellison Z Hodebourg R. Heroin seeking and extinction from seeking activate matrix metalloproteinases at synapses on distinct subpopulations of accumbens cells. Biol Psychiatry. (2021) 89:947–58. 10.1016/j.biopsych.2020.12.004 33579535 PMC8434769

[44] Johnson SW North RA. Opioids excite dopamine neurons by hyperpolarization of local interneurons. J Neurosci. (1992) 12:483–8. 10.1523/JNEUROSCI.12-02-00483.1992 1346804 PMC6575608

[45] Steffensen SC Stobbs SH Colago EE Lee R-S Koob GF Gallegos RA. Contingent and non-contingent effects of heroin on mu-opioid receptor-containing ventral tegmental area GABA neurons. Exp Neurol. (2006) 202:139–51. 10.1016/j.expneurol.2006.05.023 16814775

[46] Pettit HO Ettenberg A Bloom FE Koob GF. Destruction of dopamine in the nucleus accumbens selectively attenuates cocaine but not heroin self-administration in rats. Psychopharmacology. (1984) 84:167–73. 10.1007/BF00427441 6438676

[47] Kahlig KM Galli A. Regulation of dopamine transporter function and plasma membrane expression by dopamine, amphetamine, and cocaine. Eur J Pharmacol. (2003) 479:153–8. 10.1016/j.ejphar.2003.08.065 14612146

[48] Jhou TC. The rostromedial tegmental (RMTg)“brake” on dopamine and behavior: a decade of progress but also much unfinished work. Neuropharmacology. (2021) 198:108763. 10.1016/j.neuropharm.2021.108763 34433088 PMC8593889

[49] Parrilla-Carrero J Eid M Li H Chao YS Jhou TC. Synaptic adaptations at the rostromedial tegmental nucleus underlie individual differences in cocaine avoidance behavior. J Neurosci. (2021) 41:4620–30. 10.1523/JNEUROSCI.1847-20.2021 33753546 PMC8260244

[50] Jhou TC Good CH Rowley CS. Xu S-p, Wang H, Burnham NW, et al. Cocaine drives aversive conditioning via delayed activation of dopamine-responsive habenular and midbrain pathways. J Neurosci. (2013) 33:7501–12. 10.1523/JNEUROSCI.3634-12.2013 23616555 PMC3865501

[51] Matsui A Jarvie BC Robinson BG Hentges ST Williams JT. Separate GABA afferents to dopamine neurons mediate acute action of opioids, development of tolerance, and expression of withdrawal. Neuron. (2014) 82:1346–56. 10.1016/j.neuron.2014.04.030 24857021 PMC4072256

[52] Vogt M. The concentration of sympathin in different parts of the central nervous system under normal conditions and after the administration of drugs. J Physiol. (1954) 123:451–81. 10.1113/jphysiol.1954.sp005064 13152692 PMC1366219

[53] Babbini M Gaiardi M Bartoletti M. Dose-time motility effects of morphine and methadone in naive or morphinized rats. Pharmacol Res Commun. (1979) 11:809–16. 10.1016/S0031-6989(79)80051-X 575222

[54] Joyce EM Iversen SD. The effect of morphine applied locally to mesencephalic dopamine cell bodies on spontaneous motor activity in the rat. Neurosci Lett. (1979) 14:207–12. 10.1016/0304-3940(79)96149-4 530497

[55] Martin WR Wikler A Eades CG Pescor FT. Tolerance to and physical dependence on morphine in rats. Psychopharmacologia. (1963) 4:247–60. 10.1007/BF00408180 14048545

[56] Vasko MR Domino EF. Tolerance development to the biphasic effects of morphine on locomotor activity and brain acetylcholine in the rat. J Pharmacol Exp Ther. (1978) 207:848–58. 731435

[57] Wise RA Bozarth MA. A psychomotor stimulant theory of addiction. Psychol Rev. (1987) 94:469–92. 10.1037/0033-295X.94.4.469 3317472

[58] Holmes LJ Bozarth MA Wise RA. Circling from intracranial morphine applied to the ventral tegmental area in rats. Brain Res Bull. (1983) 11:295–8. 10.1016/0361-9230(83)90163-6 6640359

[59] Holmes LJ Wise RA. Contralateral circling induced by tegmental morphine: anatomical localization, pharmacological specificity, and phenomenology. Brain Res. (1985) 326:19–26. 10.1016/0006-8993(85)91380-0 3971145

[60] Kalivas PW Widerlöv E Stanley D Breese G Prange AJ Jr. Enkephalin action on the mesolimbic system: a dopamine-dependent and a dopamine-independent increase in locomotor activity. J Pharmacol Exp Ther. (1983) 227:229–37. 6620168

[61] Dunn KE Huhn AS Bergeria CL Gipson CD Weerts EM. Non-opioid neurotransmitter systems that contribute to the opioid withdrawal syndrome: a review of preclinical and human evidence. J Pharmacol Exp Ther. (2019) 371:422–52. 10.1124/jpet.119.258004 31391211 PMC6863456

[62] Cox B Ary M Lomax P. Dopaminergic involvement in withdrawal hypothermia and thermoregulatory behavior in morphine dependent rats. Pharmacol Biochem Behav. (1976) 4:259–62. 10.1016/0091-3057(76)90238-0 945579

[63] Acquas E Carboni E Di Chiara G. Profound depression of mesolimbic dopamine release after morphine withdrawal in dependent rats. Eur J Pharmacol. (1991) 193:133–4. 10.1016/0014-2999(91)90214-B 1646728

[64] Gramsch C Bläsig J Herz A. Changes in striatal dopamine metabolism during precipitated morphine withdrawal. Eur J Pharmacol. (1977) 44:231–40. 10.1016/0014-2999(77)90070-X 560969

[65] Gunne LM Jonsson J Fuxe K. Effects of morphine intoxication on brain catecholamine neurons. Eur J Pharmacol. (1969) 5:338–42. 10.1016/0014-2999(69)90110-1 4306742

[66] Pothos E Rada P Mark GP Hoebel BG. Dopamine microdialysis in the nucleus accumbens during acute and chronic morphine, naloxone-precipitated withdrawal and clonidine treatment. Brain Res. (1991) 566:348–50. 10.1016/0006-8993(91)91724-F 1814554

[67] Rossetti ZL Hmaidan Y Gessa GL. Marked inhibition of mesolimbic dopamine release: a common feature of ethanol, morphine, cocaine and amphetamine abstinence in rats. Eur J Pharmacol. (1992) 221:227–34. 10.1016/0014-2999(92)90706-A 1426002

[68] Fuertes G Laorden ML Milanés MV. Noradrenergic and dopaminergic activity in the hypothalamic paraventricular nucleus after naloxone-induced morphine withdrawal. Neuroendocrinology. (2000) 71:60–7. 10.1159/000054521 10644900

[69] Sloan JW Brooks JW Eisenman AJ Martin WR. The effect of addiction to and abstinence from morphine on rat tissue catecholamine and serotonin levels. Psychopharmacologia. (1963) 4:261–70. 10.1007/BF00408181 14048546

[70] Spiga S Serra GP Puddu MC Foddai M Diana M. Morphine withdrawal-induced abnormalities in the VTA: confocal laser scanning microscopy. Eur J Neurosci. (2003) 17:605–12. 10.1046/j.1460-9568.2003.02435.x 12581178

[71] Puri SK Volicer L Cochin J. Changes in the striatal adenylate cyclase activity following acute and chronic morphine treatment and during withdrawal. J Neurochem. (1976) 27:1551–4. 10.1111/j.1471-4159.1976.tb02645.x 1033988

[72] Iwamoto ET Loh HH Way EL. Circling behavior after narcotic drugs and during naloxone-precipitated abstinence in rats with unilateral nigral lesions. J Pharmacol Exp Ther. (1976) 197:503–16. 945346

[73] Rossetti ZL Melis F Carboni S Gessa GL. Dramatic depletion of mesolimbic extracellular dopamine after withdrawal from morphine, alcohol or cocaine: a common neurochemical substrate for drug dependence. Ann N Y Acad Sci. (1992) 654:513–6. 10.1111/j.1749-6632.1992.tb26016.x 1632615

[74] Shokri-Kojori E Wang GJ Volkow ND. Naloxone precipitated withdrawal increases dopamine release in the dorsal striatum of opioid dependent men. Transl Psychiatry. (2021) 11:445. 10.1038/s41398-021-01548-8 34471102 PMC8410787

[75] Diana M Pistis M Muntoni A Gessa G. Profound decrease of mesolimbic dopaminergic neuronal activity in morphine withdrawn rats. J Pharmacol Exp Ther. (1995) 272:781–5. 7853194

[76] Acquas E Di Chiara G. Depression of mesolimbic dopamine transmission and sensitization to morphine during opiate abstinence. J Neurochem. (1992) 58:1620–5. 10.1111/j.1471-4159.1992.tb10033.x 1313849

[77] Diana M Muntoni AL Pistis M Melis M Gessa GL. Lasting reduction in mesolimbic dopamine neuronal activity after morphine withdrawal. Eur J Neurosci. (1999) 11:1037–41. 10.1046/j.1460-9568.1999.00488.x 10103095

[78] Georges F Stinus L Bloch B Le Moine C. Chronic morphine exposure and spontaneous withdrawal are associated with modifications of dopamine receptor and neuropeptide gene expression in the rat striatum. Eur J Neurosci. (1999) 11:481–90. 10.1046/j.1460-9568.1999.00462.x 10051749

[79] Dockstader CL Rubinstein M Grandy DK Low MJ van der Kooy D. The D2 receptor is critical in mediating opiate motivation only in opiate-dependent and withdrawn mice. Eur J Neurosci. (2001) 13:995–1001. 10.1046/j.1460-9568.2001.01455.x 11264672

[80] Martinez D Saccone PA Liu F Slifstein M Orlowska D Grassetti A. Deficits in dopamine D receptors and presynaptic dopamine in heroin dependence: commonalities and differences with other types of addiction. Biol Psychiatry. (2012) 71:192–8. 10.1016/j.biopsych.2011.08.024 22015315 PMC3253988

[81] Wang GJ Volkow ND Fowler JS Logan J Abumrad NN Hitzemann RJ. Dopamine D2 receptor availability in opiate-dependent subjects before and after naloxone-precipitated withdrawal. Neuropsychopharmacology. (1997) 16:174–82. 10.1016/S0893-133X(96)00184-4 9015800

[82] Piepponen TP Katajamäki J Kivastik T Zharkovsky A Ahtee L. Behavioural and neurochemical sensitization of morphine-withdrawn rats to quinpirole. Pharmacol Biochem Behav. (1996) 54:787–92. 10.1016/0091-3057(95)02225-2 8853204

[83] Druhan JP Walters CL Aston-Jones G. Behavioral activation induced by D-like receptor stimulation during opiate withdrawal. J Pharmacol Exp Ther. (2000) 294:531–8. 10900229

[84] Schulz R Herz A. Naloxone-precipitated withdrawal reveals sensitization to neurotransmitters in morphine tolerant/dependent rats. Naunyn Schmiedebergs Arch Pharmacol. (1977) 299:95–9. 10.1007/BF00508644 20584

[85] Wise RA Murray A Bozarth MA. Bromocriptine self-administration and bromocriptine-reinstatement of cocaine-trained and heroin-trained lever pressing in rats. Psychopharmacology. (1990) 100:355–60. 10.1007/BF02244606 2315433

[86] Reddy PL Veeranna Thorat SN Bhargava HN. Evidence for the behavioral supersensitivity of dopamine D2 receptors without receptor up-regulation in morphine-abstinent rats. Brain Res. (1993) 607:293–300. 10.1016/0006-8993(93)91519-X 8481804

[87] Fog R. Behavioural effects in rats of morphine and amphetamine and of a combination of the two drugs. Psychopharmacologia. (1970) 16:305–12. 10.1007/BF00404736 5461437

[88] Volkow ND Wang GJ Fowler JS Logan J Gatley SJ Gifford A. Prediction of reinforcing responses to psychostimulants in humans by brain dopamine D2 receptor levels. Am J Psychiatry. (1999) 156:1440–3. 10484959 10.1176/ajp.156.9.1440

[89] Volkow ND Wang GJ Fowler JS Thanos PP Logan J Gatley SJ. Brain DA D2 receptors predict reinforcing effects of stimulants in humans: replication study. Synapse. (2002) 46:79–82. 10.1002/syn.10137 12211085

[90] Ashok AH Mizuno Y Volkow ND Howes OD. Association of stimulant use with dopaminergic alterations in users of cocaine, amphetamine, or methamphetamine: a systematic review and meta-analysis. JAMA Psychiatry. (2017) 74:511–9. 10.1001/jamapsychiatry.2017.0135 28297025 PMC5419581

[91] Sicuteri F Anselmi B Del Bianco PL. Dopamine and 5-HT supersensitivity in nonorganic central pain and in morphine abstinence: fortuitous or renal analogy? Adv Biochem Psychopharmacol. (1980) 22:523–33. 7395606

[92] De Vries TJ Schoffelmeer AN Binnekade R Raasø H Vanderschuren LJ. Relapse to cocaine- and heroin-seeking behavior mediated by dopamine D2 receptors is time-dependent and associated with behavioral sensitization. Neuropsychopharmacology. (2002) 26:18–26. 10.1016/S0893-133X(01)00293-7 11751029

[93] Glick SD Cox RD. Changes in sensitivity to operant effects of dopaminergic and cholinergic agents following morphine withdrawal in rats. Eur J Pharmacol. (1977) 42:303–6. 10.1016/0014-2999(77)90298-9 558097

[94] Schwarting RK Huston JP. Unilateral 6-hydroxydopamine lesions of meso-striatal dopamine neurons and their physiological sequelae. Prog Neurobiol. (1996) 49:215–66. 10.1016/S0301-0082(96)00015-9 8878304

[95] Iwamoto ET Loh HH Way EL. Dopaminergic-cholinergic interactions in naloxone-induced circling in morphine-dependent rats with nigral lesions. Eur J Pharmacol. (1976) 38:39–54. 10.1016/0014-2999(76)90199-0 986306

[96] Halliwell JV Kumar R. Morphine dependence and dopaminergic activity: tests of circling responses in rats with unilateral nigral lesions. Br J Pharmacol. (1980) 70:545–54. 10.1111/j.1476-5381.1980.tb09773.x 6258680 PMC2044381

[97] Glick SD Morihisa JM. Changes in sensitivity of morphine-induced circling behaviour after chronic treatment and persistence after withdrawal in rats. Nature. (1976) 260:159–61. 10.1038/260159a0 943702

[98] Lal H O'Brien J Puri SK. Morphine-withdrawal aggression: sensitization by amphetamines. Psychopharmacologia. (1971) 22:217–23. 10.1007/BF00401783 5167207

[99] Gianutsos G Hynes MD Puri SK Drawbaugh RB Lal H. Effect of apomorphine and nigrostriatal lesions on aggression and striatal dopamine turnover during morphine withdrawal: evidence for dopaminergic supersensitivity in protracted abstinence. Psychopharmacologia. (1974) 34:37–44. 10.1007/BF00421218 4856259

[100] Bieber CM Fernandez K Borsook D Brennan MJ Butler SF Jamison RN. Retrospective accounts of initial subjective effects of opioids in patients treated for pain who do or do not develop opioid addiction: a pilot case-control study. Exp Clin Psychopharmacol. (2008) 16:429–34. 10.1037/1064-1297.16.5.429 18837639 PMC3153468

[101] Agrawal A Jeffries PW Srivastava AB McCutcheon VV Lynskey MT Heath AC. Retrospectively assessed subjective effects of initial opioid use differ between opioid misusers with opioid use disorder (OUD) and those who never progressed to OUD: data from a pilot and a replication sample. J Neurosci Res. (2020) 100:353–61. 10.1002/jnr.24643 32468677 PMC8142810

[102] Lamb RJ Henningfield JE. Human d-amphetamine drug discrimination: methamphetamine and hydromorphone. J Exp Anal Behav. (1994) 61:169–80. 10.1901/jeab.1994.61-169 7513346 PMC1334405

[103] Walsh SL Sullivan JT Preston KL Garner JE Bigelow GE. Effects of naltrexone on response to intravenous cocaine, hydromorphone and their combination in humans. J Pharmacol Exp Ther. (1996) 279:524–38. 8930154

[104] Hansen ER Carvalho S McDonald M Havens JR. A qualitative examination of recent increases in methamphetamine use in a cohort of rural people who use drugs. Drug Alcohol Depend. (2021) 229:109145. 10.1016/j.drugalcdep.2021.109145 34763138 PMC8665094

[105] Baker R Leichtling G Hildebran C Pinela C Waddell EN Sidlow C. “Like Yin and Yang”: perceptions of methamphetamine benefits and consequences among people who use opioids in rural communities. J Addict Med. (2021) 15:34–9. 10.1097/ADM.0000000000000669 32530888 PMC7734765

[106] Palmer A Scott N Dietze P Higgs P. Motivations for crystal methamphetamine-opioid co-injection/co-use amongst community-recruited people who inject drugs: a qualitative study. Harm Reduct J. (2020) 17:14. 10.1186/s12954-020-00360-9 32106854 PMC7047412

[107] Kosten TA. Cocaine attenuates the severity of naloxone-precipitated opioid withdrawal. Life Sci. (1990) 47:1617–23. 10.1016/0024-3205(90)90366-Y 2250575

[108] Hermann D Klages E Welzel H Mann K Croissant B. Low efficacy of non-opioid drugs in opioid withdrawal symptoms. Addict Biol. (2005) 10:165–9. 10.1080/13556210500123514 16191669

[109] Stine SM Kosten TR. Reduction of opiate withdrawal-like symptoms by cocaine abuse during methadone and buprenorphine maintenance. Am J Drug Alcohol Abuse. (1994) 20:445–58. 10.3109/00952999409109183 7832179

[110] Preston KL Sullivan JT Strain EC Bigelow GE. Enhancement of cocaine's abuse liability in methadone maintenance patients. Psychopharmacology. (1996) 123:15–25. 10.1007/BF02246276 8741950

[111] Foltin RW Christiansen I Levin FR Fischman MW. Effects of single and multiple intravenous cocaine injections in humans maintained on methadone. J Pharmacol Exp Ther. (1995) 275:38–47. 7562574

[112] Teoh SK Mello NK Mendelson JH Kuehnle J Gastfriend DR Rhoades E. Buprenorphine effects on morphine- and cocaine-induced subjective responses by drug-dependent men. J Clin Psychopharmacol. (1994) 14:15–27. 10.1097/00004714-199402000-00003 8151000

[113] Kupchik YM Kalivas PW. The direct and indirect pathways of the nucleus accumbens are not what you think. Neuropsychopharmacol. (2017) 42:369. 10.1038/npp.2016.160 27909323 PMC5143491

[114] Albin RL Young AB Penney JB. The functional anatomy of basal ganglia disorders. Trends Neurosci. (1989) 12:366–75. 10.1016/0166-2236(89)90074-X 2479133

[115] Yawata S Yamaguchi T Danjo T Hikida T Nakanishi S. Pathway-specific control of reward learning and its flexibility via selective dopamine receptors in the nucleus accumbens. Proc Natl Acad Sci U S A. (2012) 109:12764–9. 10.1073/pnas.1210797109 22802650 PMC3412032

[116] Kupchik YM Brown RM Heinsbroek JA Lobo MK Schwartz DJ Kalivas PW. Coding the direct/indirect pathways by D1 and D2 receptors is not valid for accumbens projections. Nat Neurosci. (2015) 18:1230–2. 10.1038/nn.4068 26214370 PMC4551610

[117] Pardo-Garcia TR Garcia-Keller C Penaloza T Richie CT Pickel J Hope BT. Ventral pallidum is the primary target for accumbens d1 projections driving cocaine seeking. J Neurosci. (2019) 39:2041–51. 10.1523/JNEUROSCI.2822-18.2018 30622165 PMC6507080

[118] Roberts-Wolfe D Bobadilla AC Heinsbroek JA Neuhofer D Kalivas PW. Drug Refraining and seeking potentiate synapses on distinct populations of accumbens medium spiny neurons. J Neurosci. (2018) 38:7100–7. 10.1523/JNEUROSCI.0791-18.2018 29976626 PMC6083453

[119] Luo Z Volkow ND Heintz N Pan Y Du C. Acute cocaine induces fast activation of D1 receptor and progressive deactivation of D2 receptor striatal neurons: *in vivo* optical microprobe [Ca2+]i imaging. J Neurosci. (2011) 31:13180–90. 10.1523/JNEUROSCI.2369-11.2011 21917801 PMC3214624

[120] Heinsbroek JA Neuhofer DN Griffin WC 3rd Siegel GS Bobadilla AC Kupchik YM. Loss of plasticity in the D2-accumbens pallidal pathway promotes cocaine seeking. J Neurosci. (2017) 37:757–67. 10.1523/JNEUROSCI.2659-16.2016 28123013 PMC5296778

[121] Dobbs LK Kaplan AR Lemos JC Matsui A Rubinstein M Alvarez VA. Dopamine Regulation of lateral inhibition between striatal neurons gates the stimulant actions of cocaine. Neuron. (2016) 90:1100–13. 10.1016/j.neuron.2016.04.031 27181061 PMC4891261

[122] Gerfen CR Engber TM Mahan LC Susel Z Chase TN Monsma FJ Jr., et al. D1 and D2 dopamine receptor-regulated gene expression of striatonigral and striatopallidal neurons. Science. (1990) 250:1429–32. 10.1126/science.2147780 2147780

[123] Neve KA Seamans JK Trantham-Davidson H. Dopamine receptor signaling. J Recept Signal Transduct Res. (2004) 24:165–205. 10.1081/RRS-200029981 15521361

[124] Beaulieu JM Gainetdinov RR. The physiology, signaling, and pharmacology of dopamine receptors. Pharmacol Rev. (2011) 63:182–217. 10.1124/pr.110.002642 21303898

[125] Gallo EF Meszaros J Sherman JD Chohan MO Teboul E Choi CS. Accumbens dopamine D2 receptors increase motivation by decreasing inhibitory transmission to the ventral pallidum. Nat Commun. (2018) 9:1086. 10.1038/s41467-018-03272-2 29540712 PMC5852096

[126] Behar K. GABA synthesis and metabolism. Encyc Neurosci. (2009) 433–439. 10.1016/B978-008045046-9.01240-7

[127] Zahm DS Zaborszky L Alones VE Heimer L. Evidence for the coexistence of glutamate decarboxylase and Met-enkephalin immunoreactivities in axon terminals of rat ventral pallidum. Brain Res. (1985) 325:317–21. 10.1016/0006-8993(85)90331-2 3884089

[128] Kalivas PW Churchill L Klitenick MA. GABA and enkephalin projection from the nucleus accumbens and ventral pallidum to the ventral tegmental area. Neuroscience. (1993) 57:1047–60. 10.1016/0306-4522(93)90048-K 7508582

[129] Lu XY Ghasemzadeh MB Kalivas PW. Expression of D1 receptor, D2 receptor, substance P and enkephalin messenger RNAs in the neurons projecting from the nucleus accumbens. Neuroscience. (1998) 82:767–80. 10.1016/S0306-4522(97)00327-8 9483534

[130] Meador-Woodruff JH Mansour A Healy DJ Kuehn R Zhou QY Bunzow JR. Comparison of the distributions of D1 and D2 dopamine receptor mRNAs in rat brain. Neuropsychopharmacol. (1991) 5:231–42. 1839499

[131] Lester J Fink S Aronin N DiFiglia M. Colocalization of D1 and D2 dopamine receptor mRNAs in striatal neurons. Brain Res. (1993) 621:106–10. 10.1016/0006-8993(93)90303-5 8221060

[132] Perreault ML Hasbi A Alijaniaram M Fan T Varghese G Fletcher PJ. The dopamine D1-D2 receptor heteromer localizes in dynorphin/enkephalin neurons: increased high affinity state following amphetamine and in schizophrenia. J Biol Chem. (2010) 285:36625–34. 10.1074/jbc.M110.159954 20864528 PMC2978591

[133] Perreault ML Hasbi A O'Dowd BF George SR. The dopamine d1-d2 receptor heteromer in striatal medium spiny neurons: evidence for a third distinct neuronal pathway in Basal Ganglia. Front Neuroanat. (2011) 5:31. 10.3389/fnana.2011.00031 21747759 PMC3130461

[134] Nicoll RA Alger BE Jahr CE. Enkephalin blocks inhibitory pathways in the vertebrate CNS. Nature. (1980) 287:22–5. 10.1038/287022a0 6251377

[135] Young WS 3rd Alheid GF Heimer L. The ventral pallidal projection to the mediodorsal thalamus: a study with fluorescent retrograde tracers and immunohistofluorescence. J Neurosci. (1984) 4:1626–38. 10.1523/JNEUROSCI.04-06-01626.1984 6374062 PMC6564971

[136] Gritti I Mariotti M Mancia M. GABAergic and cholinergic basal forebrain and preoptic-anterior hypothalamic projections to the mediodorsal nucleus of the thalamus in the cat. Neuroscience. (1998) 85:149–78. 10.1016/S0306-4522(97)00573-3 9607710

[137] Mariotti M Gritti I Mancia M. The synchronizing influence of substantia innominata on the thalamus of the cat. J Sleep Res. (2001) 10:143–52. 10.1046/j.1365-2869.2001.00246.x 11422728

[138] Rada P Hernandez L Hoebel BG. Feeding and systemic D-amphetamine increase extracellular acetylcholine in the medial thalamus: a possible reward enabling function. Neurosci Lett. (2007) 416:184–7. 10.1016/j.neulet.2007.02.008 17337121

[139] Groenewegen HJ Berendse HW Haber SN. Organization of the output of the ventral striatopallidal system in the rat: ventral pallidal efferents. Neuroscience. (1993) 57:113–42. 10.1016/0306-4522(93)90115-V 8278047

[140] Sánchez-González MA García-Cabezas MA Rico B Cavada C. The primate thalamus is a key target for brain dopamine. J Neurosci. (2005) 25:6076–83. 10.1523/JNEUROSCI.0968-05.2005 15987937 PMC6725054

[141] Klitenick MA Deutch AY Churchill L Kalivas PW. Topography and functional role of dopaminergic projections from the ventral mesencephalic tegmentum to the ventral pallidum. Neuroscience. (1992) 50:371–86. 10.1016/0306-4522(92)90430-A 1279461

[142] Lewis D Sesack S. Chapter VI dopamine systems in the primate brain. Handb Chem Neuroanat. (1997) 13:263–375. 10.1016/S0924-8196(97)80008-5

[143] Grealish S Jönsson ME Li M Kirik D Björklund A Thompson LH. The A9 dopamine neuron component in grafts of ventral mesencephalon is an important determinant for recovery of motor function in a rat model of Parkinson's disease. Brain. (2010) 133:482–95. 10.1093/brain/awp328 20123725 PMC2822634

[144] Huang AS Mitchell JA Haber SN Alia-Klein N Goldstein RZ. The thalamus in drug addiction: from rodents to humans. Philos Trans R Soc Lond B Biol Sci. (2018) 373:20170028. 10.1098/rstb.2017.0028 29352027 PMC5790826

[145] Lacy RT Austin BP Strickland JC. The influence of sex and estrous cyclicity on cocaine and remifentanil demand in rats. Addict Biol. (2020) 25:e12716. 10.1111/adb.12716 30779409 PMC6916383

[146] Wade-Galuska T Galuska CM Winger G. Effects of daily morphine administration and deprivation on choice and demand for remifentanil and cocaine in rhesus monkeys. J Exp Anal Behav. (2011) 95:75–89. 10.1901/jeab.2011.95-75 21541117 PMC3014782

[147] Antons S Brand M Potenza MN. Neurobiology of cue-reactivity, craving, and inhibitory control in non-substance addictive behaviors. J Neurol Sci. (2020) 415:116952. 10.1016/j.jns.2020.116952 32534370

[148] Boyett B Wiest K McLeod LD Nelson LM Bickel WK Learned SM. Assessment of craving in opioid use disorder: Psychometric evaluation and predictive validity of the opioid craving VAS. Drug Alcohol Depend. (2021) 229:109057. 10.1016/j.drugalcdep.2021.109057 34794061

[149] Grimm JW Hope BT Wise RA Shaham Y. Incubation of cocaine craving after withdrawal. Nature. (2001) 412:141–2. 10.1038/35084134 11449260 PMC2889613

[150] Blum K Febo M Badgaiyan RD Demetrovics Z Simpatico T Fahlke C. Common neurogenetic diagnosis and meso-limbic manipulation of hypodopaminergic function in Reward Deficiency Syndrome (RDS): changing the recovery landscape. Curr Neuropharmacol. (2017) 15:184–94. 10.2174/1570159X13666160512150918 27174576 PMC5327445

[151] Blum K Baron D McLaughlin T Gold MS. Molecular neurological correlates of endorphinergic/dopaminergic mechanisms in reward circuitry linked to endorphinergic deficiency syndrome (EDS). J Neurol Sci. (2020) 411:116733. 10.1016/j.jns.2020.116733 32088516

[152] Gold MS Baron D Bowirrat A Blum K. Neurological correlates of brain reward circuitry linked to opioid use disorder (OUD): Do homo sapiens acquire or have a reward deficiency syndrome? J Neurol Sci. (2020) 418:117137. 10.1016/j.jns.2020.117137 32957037 PMC7490287

[153] Jordan CJ Cao J Newman AH Xi ZX. Progress in agonist therapy for substance use disorders: Lessons learned from methadone and buprenorphine. Neuropharmacology. (2019) 158:107609. 10.1016/j.neuropharm.2019.04.015 31009632 PMC6745247

[154] Stoops WW Rush CR. Agonist replacement for stimulant dependence: a review of clinical research. Curr Pharm Des. (2013) 19:7026–35. 10.2174/138161281940131209142843 23574440 PMC3740019

